# Lithospheric strike-slip faulting in central Tibet since 35–32 Ma and implications for the incipient Asian extrusional tectonics

**DOI:** 10.1093/nsr/nwae428

**Published:** 2024-11-28

**Authors:** Haijian Lu, Haibing Li, Zhongjin Xiang, Marco G Malusà, Chunrui Li, Zhiyong Zhang, Lin Wu, Xuxuan Ma, Jiawei Pan

**Affiliations:** SinoProbe Laboratory, Key Laboratory of Continental Dynamics of Ministry of Natural Resources, Institute of Geology, Chinese Academy of Geological Sciences, Beijing 100037, China; Jiangsu Donghai Crustal Activity in Deep Holes of the Continental Scientific Drilling National Observation and Research Station, Lianyungang 222300, China; SinoProbe Laboratory, Key Laboratory of Continental Dynamics of Ministry of Natural Resources, Institute of Geology, Chinese Academy of Geological Sciences, Beijing 100037, China; Jiangsu Donghai Crustal Activity in Deep Holes of the Continental Scientific Drilling National Observation and Research Station, Lianyungang 222300, China; SinoProbe Laboratory, Key Laboratory of Continental Dynamics of Ministry of Natural Resources, Institute of Geology, Chinese Academy of Geological Sciences, Beijing 100037, China; Department of Earth and Environmental Sciences, University of Milano-Bicocca, Milano 20126, Italy; SinoProbe Laboratory, Key Laboratory of Continental Dynamics of Ministry of Natural Resources, Institute of Geology, Chinese Academy of Geological Sciences, Beijing 100037, China; Jiangsu Donghai Crustal Activity in Deep Holes of the Continental Scientific Drilling National Observation and Research Station, Lianyungang 222300, China; State Key Laboratory of Lithospheric Evolution, Institute of Geology and Geophysics, Chinese Academy of Sciences, Beijing 100029, China; State Key Laboratory of Lithospheric Evolution, Institute of Geology and Geophysics, Chinese Academy of Sciences, Beijing 100029, China; SinoProbe Laboratory, Key Laboratory of Continental Dynamics of Ministry of Natural Resources, Institute of Geology, Chinese Academy of Geological Sciences, Beijing 100037, China; Jiangsu Donghai Crustal Activity in Deep Holes of the Continental Scientific Drilling National Observation and Research Station, Lianyungang 222300, China; SinoProbe Laboratory, Key Laboratory of Continental Dynamics of Ministry of Natural Resources, Institute of Geology, Chinese Academy of Geological Sciences, Beijing 100037, China; Jiangsu Donghai Crustal Activity in Deep Holes of the Continental Scientific Drilling National Observation and Research Station, Lianyungang 222300, China

**Keywords:** onset age, shear depth, strike-slip faulting, bimodal dyke, Lunpola basin, central Tibet

## Abstract

The onset age and depth of the central Tibet strike-slip faults are two still unresolved fundamental issues with regard to the Cenozoic tectonic evolution of central Tibet. Here we present a comprehensive dataset of geochronological, geochemical and structural data on recently discovered en-echelon dykes representing the incipient development of strike-slip faulting from the Lunpola basin in central Tibet. Our results provide evidence for mantle-derived, bimodal magmatism linked to lithospheric-scale strike-slip faulting at 35–32 Ma, and demonstrate that the central Tibet strike-slip faults are at least 20 Ma older than previously estimated (15–8 Ma). We suggest that these faults were originally connected with the lithospheric-scale Jiali and Ailao Shan-Red River shear zones exposed farther east, forming part of a straight, 2500–3000 km-long lithospheric shear zone that favored mantle upwelling and magmatic intrusion coeval with substantial uplift (1.5–2 km) of the central Tibet valley in the early Oligocene.

## Teaser

Lithospheric strike-slip faulting in central Tibet was initiated at 35–32 Ma, which are at least 20 Ma older than previously estimated (15–8 Ma).

## INTRODUCTION

**Figure 1.**
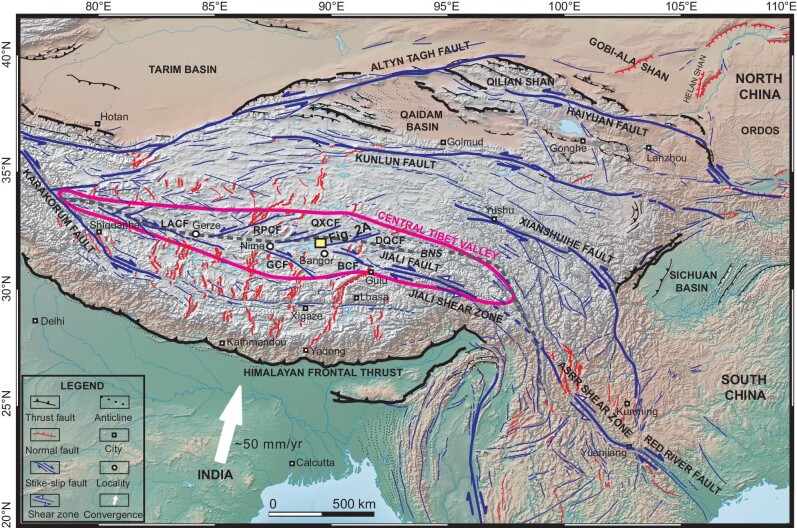
Tectonic sketch map of the Tibetan Plateau and surrounding regions, adapted from Tapponnier *et al.* [19] and location of the central Tibet valley, which spatially overlaps with the V-shaped conjugate strike-slip faults. The tectonic extrusion model suggests that the ASRR shear zone may extend westward along the Jiali shear zone and central Tibet strike-slip faults to merge with the NW-trending Karakorum fault. BNS-Bangong Nujiang suture, ASRR-Ailao Shan-Red River, LACF-Lamu Co fault, RPCF-Riganpei Co fault, GCF-Gyaring Co fault, QXCF-Qixiang Co fault, DQCF-Dongqiao Co fault, BCF-Beng Co fault.

Accurate age constraints to the elevation changes of the Tibetan Plateau are critical when evaluating the many dynamic models that may explain how the vast Tibetan Plateau was formed and is maintained, including crustal underthrusting [1] and thickening [2], escape tectonics [3], lithospheric delamination [4], slab breakoff after slab roll–back [5], lower crustal flow [6], and convective removal of the lithospheric mantle [7]. Owing to the growing application of radiometirc dating of volcaniclastic layers, an increasing number of quantitative paleoaltimetry studies are now available, which have revealed differential uplift across the various Tibetan blocks. The central Tibet valley [8,9] (Fig. 1), also referred to as the central Tibet lowland [10,11] or Bangong suture depression [12] represents one of the most striking cases, with a relatively low basin elevation (1.5–2 km) maintained until 38–29 Ma, which contrasts with the relatively high-elevation (>4 km) of the proto-plateaus established since ∼45 Ma on its northern (Qiangtang culmination-Tanggula mountain) and southern (Gangdese mountain) sides [13–15]. The central Tibet valley roughly marks the position of the late Jurassic-Early Cretaceous Bangong-Nujiang suture zone between the Qiangtang and Lhasa terranes. Geochronological, paleoaltimetry and paleontological studies collectively reveal that the valley was characterized by a subtropical lowland ‘Shangri-La’-like environment at 55–38 Ma [8–11] and was subsequently uplifted to 3–3.2 km at 26–20 Ma [16,17], or even to >4 km at 38–29 Ma [9,18]. However, the uplift mechanism of the valley remains controversial and the detachment of Asian [12] or Lhasa [9] lithosphere, compressive deformation [18], or crustal channel flow [10] have been alternatively invoked.

The central Tibet valley is spatially coincident with the V-shaped conjugate strike-slip faults that have been widely suggested to accommodate eastward extrusion of central Tibet through NW-SE striking right-lateral slip in the south, and NE-SW left-lateral slip in the north (Fig. 1). The age of activity of these strike-slip faults is vital to test the tectonic extrusion model which suggests a linkage among the Ailao Shan-Red River (ASRR) shear zone in east, the Jiali shear zone in the middle, and the central Tibet strike-slip faults in the west during the early stage of the Indian-Eurasian collision [3]. However, the ages of activity of these strike-slip faults are broadly constrained from 3–2 Ma [20] or ∼5 Ma [21] to 15–8 Ma [22–24]. It is also unclear whether the central Tibet strike-slip faults penetrate in the upper crust [25], in the lower crust [26–28], or even in the lithospheric mantle [29–31]. Additionally, unlike subduction-related postcollisional adakites and potassic-ultrapotassic volcanics in southern and northern Tibet [32,33], the late Eocene-early Oligocene (36–28 Ma) Na-rich calc-alkaline lavas and trachydacites, though less exposed in the southern Qiangtang terrane, may suggest a contribution of ascending asthenosphere to crustal thickening [34,35]. Therefore, an updated geodynamic mechanism is required to interpret the petrogenesis of these rocks.

Here, we report the discovery of bimodal magmatic rocks that are arranged according to an en-echelon pattern in the Lunpola basin of central Tibet (Fig. 1). Arrays of en-echelon fractures and veins (‘tension gashes’) can be observed in nature across a wide range of length scales. Some think that strike-slip faulting initiates with the formation of an array of en-echelon opening-mode veins and frequently occurs as a combination and an amalgamation of an en-echelon array of fractures [36,37]. Thus, an en-echelon array of fractures can be viewed as a precursor to brittle faulting [36,37]. These fracture fillings in the dykes provide a unique opportunity to investigate the postcollisional tectonics and magmatism of central Tibet. Coupled with multiple geochronological, geochemical and paleo-stress analyses, our results not only challenge some original geological understandings with respect to the timing of strike-slip faulting and uplift mechanism in the central Tibet region, but they also shed new light on the incipient Asian extrusional tectonics shortly after the onset of a hard collision between India and Eurasia.

## GEOLOGY

### Tectonic setting

The central Tibet valley, lying near the geographic center of the Tibetan Plateau and straddling the trace of the Bangong suture zone, includes the northern part of the Lhasa terrane and the southern fringe of the Qiangtang terrane [9] (Fig. 1). It roughly overlaps in position with a 200–300 km wide and 1500–1800 km long east trending zone of conjugate strike-slip faulting recognized across central Tibet [9] (Fig. 1). The NW-SE right-lateral and NE-SW left-lateral strike-slip faults are the dominant mode of deformation in central Tibet and are suggested to accommodate coeval north-south shortening with a rate of 1–2 mm/yr and east-west extension with a rate of 2–4 mm/yr [23,38]. The region was formed due to (1) the collision of the Lhasa and Qiangtang terranes following Late Jurassic-Early Cretaceous consumption of oceanic lithosphere and (2) the continued Paleocene-Oligocene contractional deformation with S-directed thrusting along its northern margin and N-directed thrusting along its southern margin [14].

This region is characterized by relatively thickened crust (∼70 km) and thin lithosphere (160–180 km) [39]. It should be noted that geophysical studies have reported a strong seismic anisotropy and pronounced low velocity and high attenuation zone beneath central Tibet, which was considered as a channel for eastward extrusion of ductile material [26,28–31]. This low velocity zone marks a region of high geothermal gradient, with temperatures ∼550°C at 5-km depth in the Lunpola basin compared to ∼150°C at the same depth outside the low-velocity zone [28]. Geophysical data, however, do not clarify whether the central Tibet strike-slip faults only affect the upper crust [25] or extend to the lower crust [26–28] or even the lithospheric mantle [29–31]. Nevertheless, the spatial correlation between active fault traces and various geophysical observations suggests that the entire lithosphere of the central Tibet valley was deformed in a coherent fashion [30,38,40] and these strike-slip fault arrays are the surface evidence of lithosphere-penetrating shear [31]. A sharp Moho offset of ∼6 km is documented in north-south geophysical sections across the Bangong-Nujuang suture zone [41–43]. It still remains unclear, however, whether these strike-slip structures are responsible for such Moho offset.

### Cenozoic paleoelevation estimates

The central Tibet valley hosts several sedimentary basins (e.g. the Bangor and Lunpola basins to the east, the Nima Basin, and the Gerze Basin to the west) where >4 km thick fluvio-lacustrine deposits were accumulated in the Cenozoic (Fig. 1). These strata contain many volcanic ash layers, fossils and paleosol nodules, spurring the extensive application of quantitative paleoaltimetry. Most studies agree that the central Tibet valley reached an elevation of 3–4 km by the Oligocene. Pioneristic stable isotopic studies indicated that the Lunpola and Nima basins have been elevated to near-modern elevations (∼4.6 km) by ∼35 Ma and 26 Ma, respectively [18,44]. However, subsequent palynological, vertebrate fossils and isotopic studies consistently place the Lunpola basin at 3–3.2 km elevation during the late Oligocene to early Miocene time span [16,17,45]. The occurrence of subtropical fossil plants and fishes suggests ∼1 km surface elevation for the Nima and Lunpola basins in the late Oligocene [46]. Recent multimethod isotopic studies suggest that the Lunpola basin had reached a surface elevation of >3 km, or even >4 km at 38–29 Ma [9,47].

### Mesozoic-Cenozoic strata in the Lunpola basin

**Figure 2.**
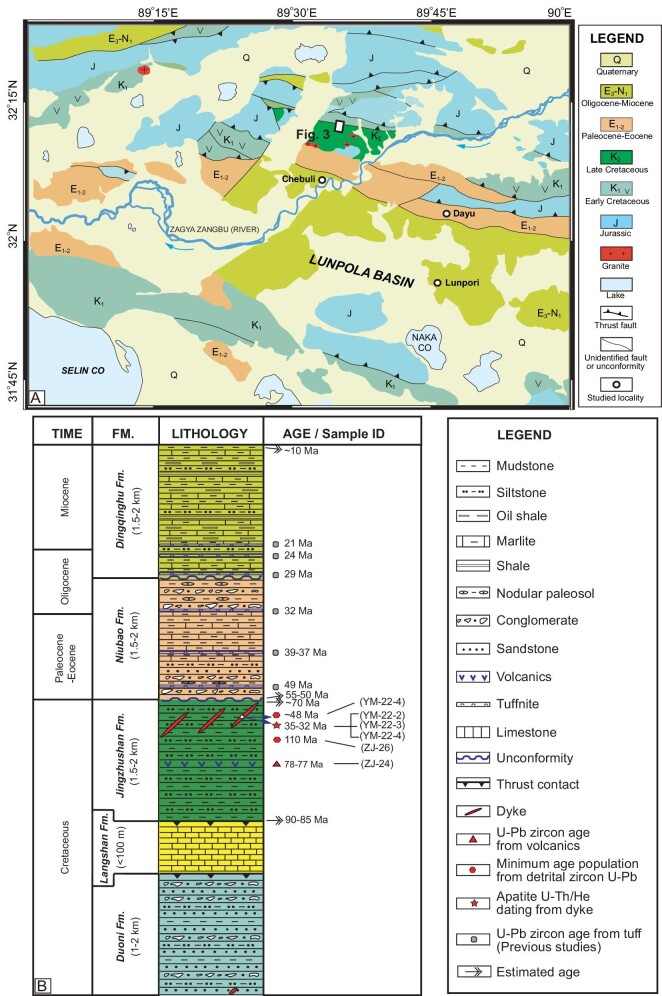
Geological map (A), modified from GSJL [49,50], and integrated Mesozoic-Cenozoic stratigraphic column (B) of the Lunpola basin in central Tibet.

**Figure 3.**
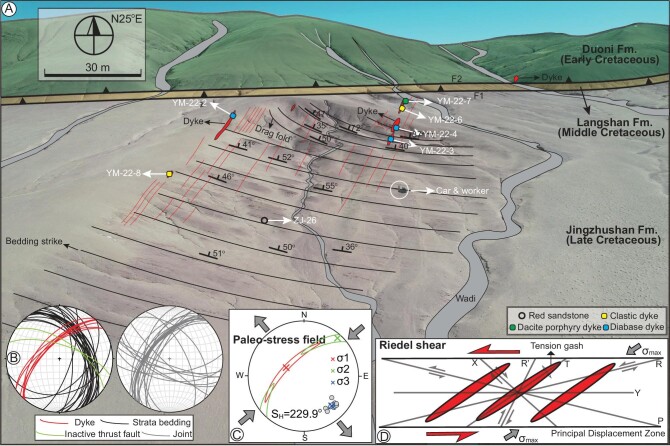
Section description, sample locations, orientations and paleostress results from the Lunpola Basin. (A) Unmanned aerial vehicle photograph of studied section superimposed by locations of samples and simplified geological map. The inset plot (B) shows the orientation data of the Jingzhushan Fm. red beds, dykes, two thrust faults, and joints. The inset plots (C–D) indicate stress analysis results for dykes and Riedel shear model, respectively. Outward- and inward-pointing arrows represent extension and compression, respectively. SH indicates the maximum horizontal compressive stress vector. The field orientation data suggest that the dykes were intruded under the influence of NE-SW–compressional stress and NW-SE–extensional stress.

Stratigraphic units in the Lunpola basin generally have Cretaceous and Cenozoic ages (Fig. 2). The Cretaceous units consist of Lower Cretaceous volcaniclastic sandstones, siltstones and conglomerates of the Duoni Formation (Fm.), Mid-Cretaceous shallow-marine limestones of the Langshan Fm. and Upper Cretaceous terrigenous red beds of the Jingzhushan Fm. [48]. The Duoni Fm. is in thrust-fault contact with the underlying Langshan Fm., which is also in thrust-fault contact with the underlying Jingzhushan Fm. (Figs 2, 3, and Fig. S1). The 1–2 km-thick Duoni Fm. displays a penetrative cleavage, tight upright to overturned folds, and transposed bedding (Fig. S1). The Langshan Fm., with a thickness not exceeding 100 m, consists of white massive, cleaved limestones (Fig. S1) and represents the youngest marine strata [48]. The Jingzhushan red beds experienced mild folding and chiefly consist of red siltstones and sandstones and minor interbedded volcanics (Fig. S1).

The 3–4 km-thick Cenozoic sedimentary sequences include the Niubao and Dingqinghu Fms (Fig. 2). The Niubao Fm. overlies unconformably the Upper Cretaceous Jingzhushan Fm. and includes grayish-green to grayish-white siltstones, mudstones, marls, and tuffs with fossil leaves and fishes, sandwiched between upper and lower red conglomerates, sandstone, siltstones, and paleosols. The depositional age of the Niubao Fm. was determined to 55–29 Ma based on multiple well-dated volcanic ash layers [9,10]. There is a low-angle unconformity between the Dingqinghu and Niubao Fms. The Dingqinghu Fm., with a maximum thickness of 1.5–2 km, consists primarily of gray to yellowish-gray fine-grained sandstones, siltstones, mudstones, shales, marls and several tuff layers. The depositional age of the Dingqinghu Fm. was constrained to 29–10 Ma [9,16,17,47].

### Dyke description

**Figure 4.**
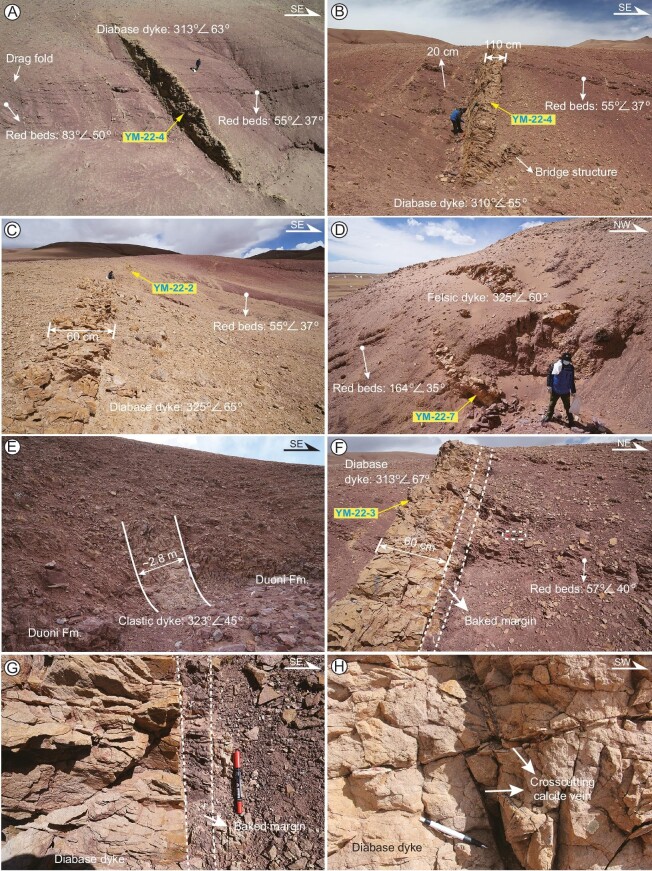
Field photos of the dykes and Jingzhushan Fm. red beds in the Lunpola Basin. The dykes consist of three thick (>60 cm) layers and the pervasively distributed thin (<20 cm) layers which have the same orientation and are both arranged in an en-echelon pattern. (A–G) Crosscutting relationships between the dykes and the folded Jingzhushan Fm. (F, G) Dark chilled margins observed at the contacts of the dykes with the Jingzhushan red beds. (H) Crosscutting calcite veins in the diabase dyke. The yellow base fonts represent sampling sites.

Due to differential exhumation, the dykes crop mainly out in the folded Jingzhushan Fm. red beds and subordinately in the Duoni Fm. (Figs 3, 4). The Jingzhushan red beds dip to the ENE with angles of 30–75° (Supplementary dataset 1). As a result of the emplacement of the dykes, the Jingzhushan red beds exhibit locally tight upright to overturned structures and drag fold (Fig. 3 and Fig. S1). The dykes dip to the NW with angles of 45–67° (Supplementary dataset 1). The dykes experienced minor tectonic disturbance as evidenced by cross-cutting calcite veins in the diabase dyke (Fig. 4H). Most of the dykes exhibit regular and straight contacts with the host rock and sharp and thin tips (Fig. 4). Because the dykes cut across the folded structures of the country rocks, it can be considered as a typical post-tectonic intrusion. Dark chilled margins observed at the contacts of the dykes with the Jingzhushan red beds indicate that they are primary emplacement features (Fig. 4F and G).

The exposed dyke segments range from several to 40-m long and their thickness does not commonly exceed 1 m (5–110 cm). The dykes can be divided into two groups: one with a thickness of >60 cm and the other ≤20 cm. Despite limited exposure, three thick dykes (>60 cm) appear arranged in an en-echelon pattern and sometimes bridges develop between segments (Fig. 4B). These thin dykes have the same orientation as those thicker ones and are pervasively distributed in the Jingzhushan red beds.

The dykes show a bimodal distribution in composition, with half diabase and half dacite porphyry and clastic dykes according to field and petrographic observations (Fig. 4 and Fig. S2) and geochemical analyses below. The diabase dykes underwent low- to moderate alteration under the microscope (Fig. S2).

## RESULTS

### Zircon U-Pb ages

Representative cathodoluminescence (CL) images of zircon grains of all samples are shown in Fig. S3. All zircon U-Pb ages are presented in Figs S4, S5 and in the Supplementary dataset 2. The crystalline age of the interbedded volcanic rock (ZJ-24) in the Jingzhushan Fm. red beds is ∼77 Ma. The detrital ages of the red sandstone (ZJ-26) in the Jingzhushan Fm. red beds range from 2827 Ma to 109 Ma, with the youngest U-Pb age population being 119–109 Ma. The ages of the diabase sample (YM-22-4) vary from 3237 Ma to 38 Ma, indicating a significant contribution of detrital origin. The youngest population of 54–38 Ma can be used to constrain the emplacement age of the diabase dykes. The ages of the clastic dykes (YM-22-6 and YM-22-8) range from 3218 Ma to 74 Ma, with the youngest population being 78–74 Ma. The age range of the dacite porphyry dykes (YM-22-7) is between 1709 Ma and 72 Ma, with the dominant youngest population being 81–72 Ma.

The depositional age of the Jingzhushan Fm. is broadly constrained to ∼90–70 Ma by the age of the interbedded volcanic rock (ZJ-24, ∼77 Ma) in the middle part of the Jingzhushan Fm., the youngest U-Pb age population of detrital zircons in the sandstone (ZJ-26, 120–107 Ma) and the approximate thickness (1.5–2 km) of the Jingzhushan Fm. red beds (Fig. 2 and Fig. S5). The emplacement age of the diabase dykes is determined to be younger than ∼40 Ma by the youngest U-Pb zircon age population (54–38 Ma) of the diabase sample YM-22-4. The intrusive ages of the clastic and dacite porphyry dykes are constrained to be younger than 78–72 Ma by the youngest U-Pb age population of the samples YM-22-6, YM-22-7 and YM-22-8 (Fig. S5). These 78–72 Ma ages are consistent with the age of the interbedded volcanic rock (ZJ-24, ∼77 Ma) in the Jingzhushan Fm. and the Late Cretaceous Madeng volcanics (73 Ma) in the Lunpola basin [9]. These identical ages from various geological units with different formation ages likely imply the existence of inherited zircons, which is often observed in anatectic melts and S-type granitoids that have abundant zircon in the melt source area [51].

### Apatite (U-Th)/He ages

**Figure 5.**
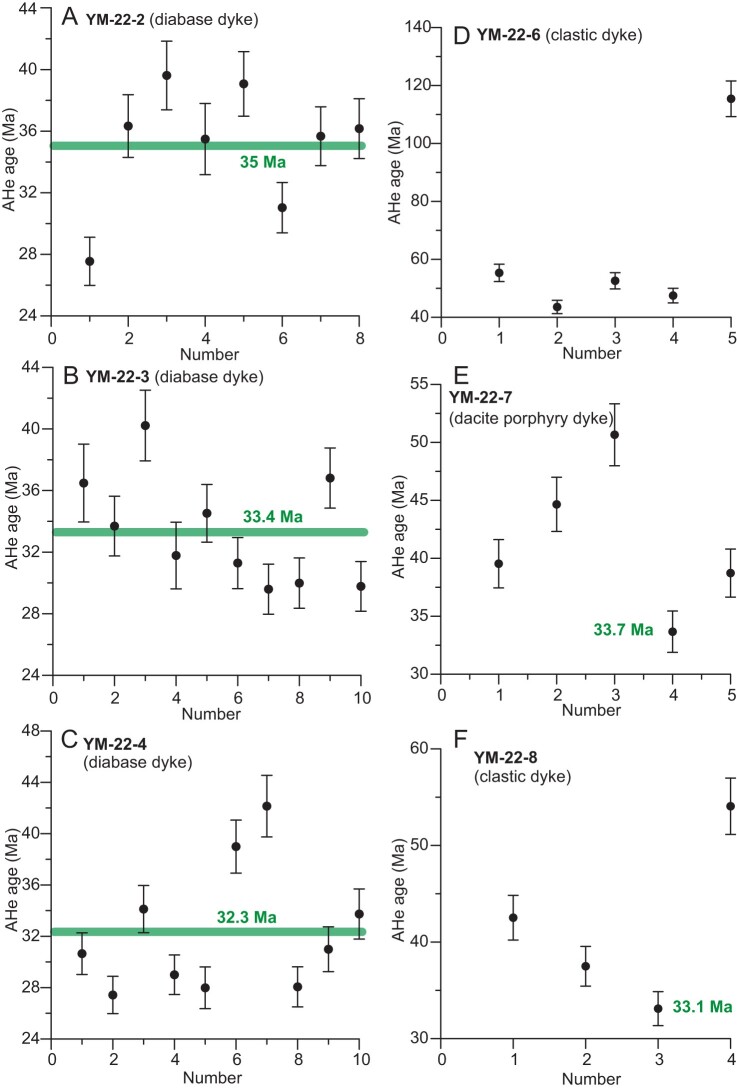
AHe ages for the diabase (YM-22-2, YM-22-3 and YM-22-4), dacite porphyry (YM-22-7) and clastic dyke (YM-22-6 and YM-22-8) samples from the Lunpola Basin.

The results of AHe thermochronology are summarized in Supplementary dataset 3 and illustrated in Fig. 5. The 3 diabase samples, each with 8–10 AHe ages from replicated measurements, have restricted ages that range from 42 Ma to 27 Ma, with a mean at 35–32.3 Ma. As no positive correlations of spherical equivalent grain radius (Rs) and/or eU with AHe age was evident (Fig. S6), we suggest that the dyke samples have experienced either rapid cooling when passing through the AHe partial resetting zone or that other factors, such as radiation damage, U–Th zoning, or fluid inclusions, have not affected the AHe ages [52,53]. This consistency in age not only corroborates the reliability of our AHe data, but also supports the onset of rapid cooling by ∼35–32 Ma.

The clastic (YM-22-6 and YM-22-8) and dacite porphyry (YM-22-7) dyke samples, however, yielded more dispersed single-grain AHe dates that range from 115 Ma to 33 Ma, with the majority of ages being 80–40 Ma. These Late-Cretaceous-Early Cenozoic ages are consistent with other AHe dates from central Tibet, indicating minimal exhumation in the region since ∼45 Ma [54,55]. Of all these ages, two youngest ages (33.1 Ma and 33.7 Ma) are in agreement with this coherent pattern of ages in the diabase samples (Fig. 5), suggesting a shared cooling history. The date dispersion in the clastic and dacite porphyry samples may result from a contribution of country rocks to the dyke samples, which is also demonstrated by the zircon U-Pb ages (Fig. S5), apatite morphology (Fig. S7) and photomicrographs (Fig. S2) of the clastic and dacite porphyry dykes. In brief, all dykes may have experienced a synchronous rapid cooling at ∼35–32 Ma (Fig. 2).

### Whole-rock and mineral compositions

Whole-rock major- and trace-element and Sr–Nd isotopic data of the dykes are presented in Supplementary dataset 4. The dykes show a bimodal composition based on their petrographic features and SiO_2_ contents. The mafic dykes are characterized by low SiO_2_ (46.08–52.59 wt.%), Na_2_O and K_2_O (Na_2_O + K_2_O = 3.19–5.48 wt.%) contents and Mg^#^ (0.44–0.55) values, high Fe_2_O_3_ (8.97–10.71 wt.%), MgO (4.02–5.55), TiO_2_ (1.07–1.32 wt%) and CaO (12.10–16.75 wt.%) contents. The mafic dykes plot into the gabbro field of the TAS diagram (Fig. S8). In the Harker diagrams, TiO_2_, Al_2_O_3_, Fe_2_O_3_ and P_2_O_5_ negatively, and Na_2_O positively correlate with SiO_2_ (Fig. S9), which means the magma may have undergone fractional crystallization of pyroxene, apatite and plagioclase. The mafic dykes are enriched in light rare earth elements (LREEs) and have remarkable differentiation between LREEs and HREEs (heavy rare earth elements) with (La/Yb)_N_ ranging from 31.1 to 33.8, and total REE contents up to 381 times chondrite. All samples have relatively steep right-dipping curves in the chondrite-normalized REE patterns without negative Eu anomaly (δEu = 0.8–0.9) and are enriched in large ion lithophile elements (LILEs), including Rb, Ba, Th and U, and LREE, but with negative K and Sr anomalies in primitive mantle (PM)-normalized trace element spider patterns (Fig. S10). The negative Sr anomalies without Eu anomaly may be due to alteration effects. They are depleted in high field strength elements (HFSEs) with strongly negative Nb, Ta, Zr and Hf anomalies, similar to those of subduction-related rocks. The (^87^Sr/^86^Sr)_i_ ratios and εNd(t) values have been calculated at 35 Ma based on the rapid cooling ages of apatite U-Th/He dating. The mafic dykes display relatively high (^87^Sr/^86^Sr)_i_ ratios (0.707853–0.708669) and homogeneous εNd(t) values (−2.1 to −2.2) (Fig. S8).

The felsic dykes have relatively high SiO_2_ (66.05–70.47 wt.%), Al_2_O_3_ (16.33–18.79 wt.%) and Na_2_O (7.96–9.66) contents, low MgO (0.11–0.22 wt.%) and Fe_2_O_3_ (2.86–3.50 wt.%) contents. The felsic dykes plot into granite areas in the TAS diagram (Fig. S8). They are characterized by high Al_2_O_3_ and Na_2_O with A/CNK values ranging from 1.05–1.22, and belong to peraluminous types (Fig. S11). In the Harker diagrams, the contents of Al_2_O_3_, Na_2_O and P_2_O_5_ decrease with that of SiO_2_ (Fig. S9), which implies that the magma may have undergone plagioclase, apatite and zircon fractional crystallization.

The felsic dykes are also enriched in LREEs and have a slight differentiation between LREE and HREE with (La/Yb)_N_ ranging from 24.9 to 29.2, and total REE contents are 178.3–245.3 ppm. All samples have relatively flat heavy REEs (HREEs) in the chondrite-normalized REE patterns with (Dy/Yb)_N_ values of 1.2–1.3 and weakly negative Eu anomaly (Eu = 0.8–0.9) and are enriched in large ion lithophile elements (LILEs), including Th and U, and LREE, but with variable negative Rb, Ba and K anomalies in primitive mantle (PM)-normalized trace element spider patterns (Fig. S10). The negative Sr anomalies without Eu anomaly may be ascribed to the alteration effect. They are depleted in high field strength elements (HFSEs) with strongly negative Nb, Ta and Ti anomalies. The negative Ti anomaly may result from the Ti-bearing mineral fractional crystallization, i.e. sphene and Ti-Fe oixdes. The felsic dykes display similar (^87^Sr/^86^Sr)_i_ ratios (0.7074–0.7088) to those of the mafic dykes and have εNd(t) values between −1.7 and −1.9 (Fig. S8).

### Paleo-stress field

The orientation data of dykes record a strike-slip stress with an almost NE-SW inclined σ1 axis, NE-SW horizontal σ2 axis and SE-NW less inclined σ3 axis, and the stress ratios (ø) = 0.63, indicating that the dykes were intruded under the influence of NE-SW–compressional stress and NW-SE–extensional stress (Fig. 3). The direction of maximum horizontal compressive stress (S_H_) is 229.9^o^.

## DISCUSSION

Below we will discuss the detrital composition, age, genesis of the dykes and their implications for the onset of the central Tibet strike-slip faulting and uplift mechanism of the central Tibet valley and tectonic escape hypothesis.

### A considerable amount of clastic intrusion into the dykes

The clastic dyke samples (YM-22-6 and YM-22-8) show a similar textural feature to that of normal sandstone under the microscope (Fig. S2). Combined dating analyses of zircon U-Pb and apatite (U-Th)/He for clastic dykes also indicate sandstone-like detrital age spectra (Fig 5 and Fig. S5). Interestingly, zircon U-Pb analysis of the diabase dyke sample (YM-22-4) likewise shows the contribution of detrital origin. These zircon grains of the diabase dyke are relatively small (<100 µm), but almost equal in size (Fig. S3), likely suggesting a significant input of siltstone. The zircon grains in the diabase sample were most likely sourced from country rocks and no new magmatic zircon was produced because there is an insufficient amount of time for crystallization differentiation to crystallize zircons [51]. In addition, two dating analyses of zircon U-Pb and apatite (U-Th)/He for the dacite porphyry dyke sample (YM-22-7) show a minor detrital origin (Fig. 5 and Fig. S5).

Therefore, there is a large amount of clastic intrusion into the dykes (Fig. S12). Sandstone intrusion is a common and widespread phenomenon and frequently develops in tectonically active settings where applied tectonic stresses facilitate development of high fluid pressures within the sediments [56,57]. Certain studies show that as it is increasingly difficult to liquefy sediment at greater depths, clastic dyke can only migrate a maximum of ∼10 m vertically from their source bed during earthquake-induced liquefaction [56]. Therefore, clastic dyke is commonly derived from superficial sedimentary layers (Fig. S12). This observation may be well used to explain why these detrital apatite grains in the clastic and dacite porphyry dykes were not completely overprinted and reset by magmatic heat during coincident intrusion of these dykes (Fig. 5).

### Timing of dyke emplacement

Except for the AHe ages of the diabase dykes, other data, including the U-Pb and AHe ages of the clastic and dacite porphyry dykes and the U-Pb ages of the diabase dykes, show somewhat scattered single grain ages, with the youngest age population (54–38 Ma) that constrains the emplacement age of the dykes. As U-Th/He dating can be applied as a geochronometer to constrain the age of a volcanic deposit [58], the coherent AHe ages (35–32 Ma) of the dykes offer a precise constraint on the emplacement age of the intrusive rocks.

In addition, based on certain indistinguishable features (e.g. grayish yellow color and same dyke attitude) among these three dykes (Fig. 4), it is concluded that they were emplaced simultaneously following fracture opening (Fig. S12). Therefore, stratigraphic contact relationships, coupled with detailed structural and geochronological data indicate that the late Cretaceous (90–70 Ma) Jingzhushan red beds were firstly folded by the thrust faults (F1 and F2) between 65 and 50 Ma, likely in response to the Indian-Eurasian collision and then intruded by bimodal magmatic rocks at 35–32 Ma (Fig. S13).

### Genesis of the Lunpola bimodal volcanic rocks

The mafic dykes have relatively low Cr (205–303 ppm), Co (29.6–37.8 ppm) and Ni (117–164 ppm) contents that imply none of them represents a primary magma. Samples with high MgO contents (>5 wt.%) do, however, have low SiO_2_ content (<50 wt.%) and high Nd(t) (up to −2.2) values, indicating that they were generated by partial melting of a mantle source. The Nb/Ta ratios (19.5–21.3) of the mafic dykes are much higher than values for the crust (10.91) but they are close to those for the mantle (17.39–17.78), which also supports their mantle origin. The mafic dykes have relatively low Mg^#^ (0.44–0.55) values which imply that they were derived from a mantle source metasomatized by crustal materials, like the source of basalts in volcanic arc settings (Fig. S11). This is also supported by the Nd(t) (−2.4∼−2.0) values that are identical to those of the Linzizong volcanic rocks (Fig. S8), which were commonly interpreted to be formed in subduction-related arc settings [34,59]. The mafic dykes have relatively high Ta/Yb (0.37–0.41) and Th/Yb (5.01–5.84) ratios which indicates that they were formed in an arc setting.

The felsic dykes are characterized by low Mg^#^ (0.07–0.11) values and high SiO_2_ contents, which are typical of crustal-derived magmas. They have high alkaline contents (Na_2_O + K_2_O = 8.18–10.19 wt%), ratios of (Na_2_O + K_2_O)/(FeOt + MgO + TiO2) and low Cao/(FeOt + MgO + TiO2), indicating a metagreywacke source (Fig. S11). In the Rb vs (Y + Nb) diagram (Fig. S14), all the samples plot in the field of volcanic arc granitoids (VAGs), implying that they were formed in an arc setting.

Field observations and dating results collectively show that the mafic and felsic dykes were emplaced synchronously. The major element compositions for all the rocks show no obvious trend in variation (Fig. S9), which is typical of these bimodal volcanic rocks that are considered to occur in extensional setting [60]. As mentioned above, the trace elements support that both the mafic and felsic dykes originated in an arc setting. Therefore, we propose that the mafic dykes were derived from partial melting of a metasomatized mantle source with subducted slab component. The felsic dykes were generated by continuous fractional crystallization originating from the basaltic parents with a significant contribution from crustal contamination.

### The onset of the central Tibet strike-slip faulting since ∼35 Ma

En echelon veins, as incipient shear fractures, mostly formed originally at angles <45^o^ with respect to shear zone walls and progressively rotate within the shear zones [61]. The lithospheric-scale fractures formed by strike-slip faulting would open, facilitating the ascent of magma from deep sources to shallow depths. Magma ascent through a composite system of tensional and shear fractures in an overall strike-slip deformation regime (horizontal σ1 and σ3) along the Bangong-Nujiang suture zone is in line with the dynamic models of fracture propagation in the lithosphere suggested by Hill (1977) [62] and Shaw (1980) [63]. The identification of the paleo-stress field enables us conclude that the formation of the dykes was controlled by NE-SW–compressional stress and NW-SE–extensional stress, as is evident judging by the Riedel shear which show right-stepping fractures on left-lateral shear (Fig. 3). This argument gains support from the observation that these NNE striking normal faults along the Yibug Caka and Bue Co are linked with the left-slip faults in the Qiangtang terrane (Taylor *et al.*, 2003) [23]. As the dykes extend northeastward to cut across the Duoni Fm. (Figs 3, 4), maybe as well as the Langshan Fm. and the WNW-striking thrust faults, the ENE-striking sinistral strike-slip faults are the sole structures that dominate the development of the dykes.

The 35–32 Ma ages may thus represent the onset of activity of the central Tibet strike-slip faults. These ages are at least 20 Ma older than previously estimated 15–8 Ma ages based on the assumption that the strike-slip faults in central Tibet developed simultaneously with the north trending rift system in the Lhasa and Qiangtang blocks [20,22–24]. This observation, in combination with the fact that these rifts in the Lhasa and Qiangtang blocks do not cross the Bangong-Nujiang suture zone, collectively indicate that there is no genetic relationship between the north trending rift system in the Lhasa and Qiangtang blocks and the central Tibet strike-slip fault system. In addition, the much older onset age (35–32 Ma) and the relatively low slip rate (<4 mm/yr) [64] and low magnitude of surface displacement (<20 km) [20] for the central Tibet strike-slip faults may reflect a non-steady state behavior of the faults.

### Uplift mechanism of the central Tibet valley

**Figure 6.**
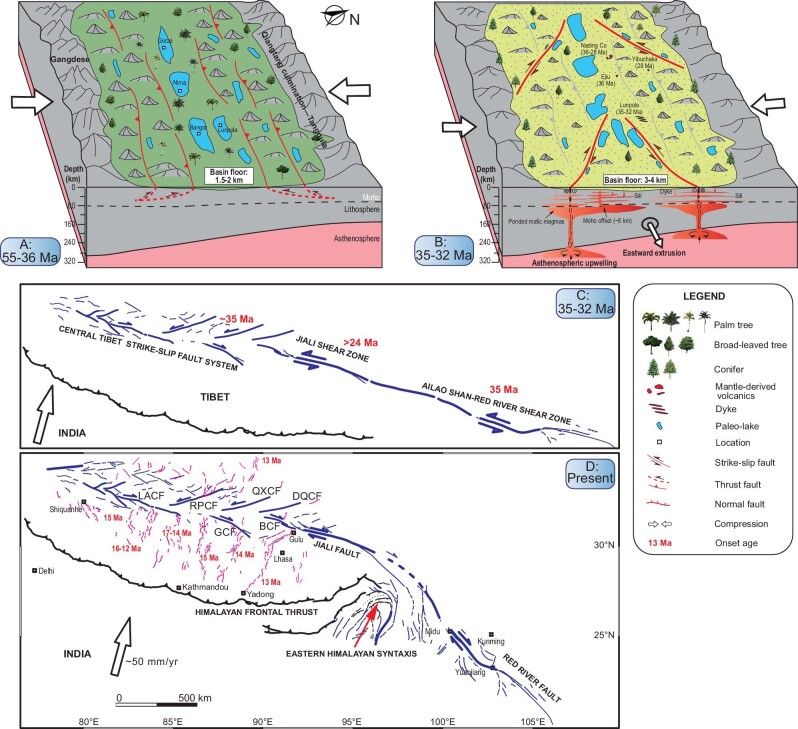
Schematic two-stage evolution models for the central Tibet valley (A–B) and a hypothesized 2500–3000 km-long major strike-slip shear zone (C, D). The block models demonstrate tectonic, topographic and biotic evolution of the central Tibet valley from 55–36 Ma (A) to 35–32 Ma (B) and the proposed geodynamic mechanism. The nearly straight major strike-slip shear zone was extending roughly from the Ailao Shan-Red River shear zone in the east, to the Jiali shear zone in the middle, and to the central Tibet strike-slip faults in the west at 35–32 Ma (C), which bend progressively to its present shape (D) due to the indentation of the Indian lithosphere in the region around the eastern Himalayan syntaxis.

Besides the bimodal magmatic rocks in the Lunpola basin, late Eocene-early Oligocene Na-rich calc-alkaline lavas (36–28 Ma) [34] and trachydacite (36 Ma) [35] in several localities near the Nima county, southern Qiangtang terrane indicate also a primitive mantle source and magmatic differentiation or melting of hydrous mafic materials, respectively. Since strike-slip fault systems have long been implicated as efficient pathways through which magma is transported, stored and eventually erupted at the earth surface in a variety of tectonic settings [62,63,65,66], we suggest that these mantle-derived magmatic rocks within the central Tibet valley are emplaced through fracture networks produced by strike-slip deformation (Fig. 6A, B). The presence of mafic magmatic rocks suggests that the central Tibet strike-slip faults are lithospheric-scale [29–31,38,40], rather than upper crust-scale [25] or lower crust-scale structures [26–28]. This observation provides support to the argument that the central Tibet lithosphere was deformed coherently, thus arguing for a lithospheric source of anisotropy [30,31,38,40,67], and a horizontal basal shear driven by an asthenospheric eastward flow.

Given the spatio-temporal coincidence, we postulate that the central Tibet strike-slip faults and accompanying mantle upwelling and magmatic intrusion were primarily responsible for the 1.5–2 km uplift of the central Tibet valley during 36–29 Ma (Fig. 6A, B). Large igneous intrusions emplaced into the crust and a hot upwelling mantle have long been believed to produce substantial surface uplift when magma input exceeds the rate of extension [68].

The mantle upwelling may lead directly to the occurrence of vertical steps in the Moho of the central Tibet valley due to rift-induced magma injection [42]. This uplift model implies that eastward extrusion and surface uplift of central Tibet took place at the same time. Due to the lack of geological and geophysical constraints, other uplift models—including the convective removal and crustal channel flow models—remain conjectural. The compressive deformation model does not gain support from the low-temperature thermochronologic data which indicate minimal exhumation since ∼45 Ma in central Tibet [54,55]. These contemporary volcanic fields in the Nading Co and Lunpola basin are flat-lying, or undeformed [34], indicating insignificant compressional deformation since 36–28 Ma. The structural analyses in the Lunpola basin also show that strike-slip faults, rather than thrust faults, are the dominating structures in the Niubao and Dingqinghu strata (Fig. S15).

### A major strike-slip shear zone connecting the ASRR shear zone and the central Tibet strike-slip faults

The eastward tectonic extrusion of the Indochina block was accommodated by the NNW-trending sinistral ASRR shear zone, a long (>1000 km), narrow (<20 km) zone of amphibolite-facies gneiss and mylonite, extending from Tibet to the South China Sea with a displacement of 700 ± 200 km between 35 and 17 Ma, though the major shear zone reversed its motion sense as a right‐lateral fault since ∼10 Ma with ∼40 km displacement [69–74]. The Jiali shear zone, similar to the ASRR shear zone, had reversed its motion sense from sinistral to dextral strike-slip fault during the Late Miocene [75]. The sinistral strike-slip along the Jiali shear zone commenced during or prior to Late Oligocene (∼24 Ma or even earlier), which can be correlated with the Oligocene sinistral movement along the ASRR shear zone [75]. Geochronological data coupled with structural investigations led the extrusion model to link the ASRR shear zone to the NWW-trending fault systems in Tibet (e.g. Jiali shear zone), indicating that the major shear zone may continue somehow to the northwest [3,70,71,74,75]. Block reconstruction of Asia suggest that the ASRR shear zone may extend westward along the Bangong suture to merge with the NW-trending Karakorum fault [76,77]. Armijo *et al.* (1989) [22] also recognized that this system of en-echelon NW-SE right-lateral faults in central Tibet can extend from the Karakoram Fault in the west, to at least the eastern termination of the Jiali fault zone near the eastern Himalayan syntaxis, accommodating the eastward movement of the Qiangtang terrane relative to the Lhasa terrane (Fig. 1).

The paleo-stress field reconstruction based on the field orientation data of the dykes suggests that central Tibet was controlled tectonically by a combination of NE-SW–compressional stress and NW-SE–extensional stress, which is consistent with the observations above. Since the central Tibet strike-slip faults may also initiate at ∼35 Ma, it implies that these strike-slip faults in central Tibet most likely connected with the ASRR shear zone. Therefore, our study supports the existence of an almost 2500–3000 km-long strike-slip shear zone (Fig. 6C) extending roughly from the ASRR shear zone in the east, to the Jiali shear zone in the middle, and to the central Tibet strike-slip faults in the west [3,70,71,74,76,77]. The displacement along this major shear zone is largest (700 ± 200 km) in the center (the northwest portion of the ASRR shear zone) and decrease markedly toward its eastern and western ends [78].

At the early stage of the collision, the Bangong suture and the ASRR shear zone behaved as a weak inherited heterogeneity allowing for the localization of strain along this belt. Its shape was nearly linear or slightly curved as suggested by Replumaz and Tapponnier (2003) [77] and Replumaz *et al.* (2014) [76] in their reconstruction models. Subsequently, due to the continued northward push of the eastern Himalayan syntaxis and related clockwise rotation of southeast Tibet, this shear belt bends progressively to its present shape (Fig. 6D). In the bending process of this shear zone, the central Tibet strike-slip faults may gradually become inactive. The bending of this shear belt may be well used to account for inconsistent behavior of the central Tibet strike-slip faults over its prolonged tectonic evolution. In conclusion, our study, to a certain extent, seems to support the escape tectonics model [3].

## MATERIALS AND METHODS

We collected one red sandstone sample (ZJ-26) from the upper part of the Jingzhushan Fm. and one interbedded volcanic rock (ZJ-24) from the central part of the Jingzhushan Fm. for zircon U-Pb dating (Figs 2, 3). We also collected three diabase dyke samples (YM-22-2, YM-22-3 and YM-22-4) and two clastic dyke samples (YM-22-6 and YM-22-8) and one dacite porphyry dyke sample (YM-22-7) for various analyses (Figs 2, 3), including thin section, apatite U-Th/He and zircon U-Pb dating, whole-rock major and trace element and Sr–Nd isotope analyses. Apatite fission track and U-Pb dating for these diabase samples are unavailable due to either low U or high common Pb concentrations [51]. We did not observe baddeleyite in the diabase samples likely because of low Zr concentrations. The Supplementary Data provides more details for these four kinds of experimental measurement.

### Zircon U-Pb dating

Zircon grains were obtained according to standard mineral separation techniques. Approximately 300 zircon grains for each sample were mounted in epoxy resin and polished to acquire a smooth flat internal surface. The reflected, transmitted light microscopy, and cathodoluminescence (CL) imagery were collectively used to identify ideal grains for zircon U-Pb analysis (Fig. S3). We dated 24 zircon grains for the volcanic rock sample (ZJ-24), and 73 and 99 zircon grains for the red sandstone ZJ-26 and the diabase dyke YM-22-4, respectively. As the zircon grains of the dyke samples YM-22-6, YM-22-7 and YM-22-8 exhibit mixed sources with significant input from country rocks, we dated 84, 117 and 86 zircon grains for them, respectively.

### Apatite U-Th/He dating

Apatite (U-Th)/He dating was conducted in ^40^Ar/^39^Ar and (U-Th)/He geochronology laboratory, Institute of Geology and Geophysics, Chinese Academy of Sciences (IGGCAS). Apatite grains were obtained using the standard rock crushing and heavy liquid separation processes. These relatively euhedral apatite grains free of visible inclusions and internal fractures and with half-widths >40 μm were hand-picked under a binocular microscope. There are 3–5 grains from each of the dyke samples. The diabase samples have been dated twice in order to evaluate the replicability of the results.

### Whole-rock geochemistry and Sr-Nd isotopic measurement

The analyses of whole-rock major, trace elements, and Sr-Nd isotopic measurements were carried out at Nanjing FocuMS Technology Co. Ltd (Nanjing, China). Fresh rock chips were powdered to <200 μm for major and trace elemental concentrations and radiogenic isotope analyses.

### Paleo-stress field

The orientations of dykes were used to reconstruct the paleo-stress field at the time of magma intrusion using the method of Yamaji and Sato [79] and the ‘GArcmB’ software [80]. The method fits a linear combination of Bingham distributions to the poles to dykes through a real-coded genetic algorithm. The σ1, σ2 and σ3 axes represent maximum-, intermediate-, and minimum-concentration axes of a Bingham distribution, respectively [81]. The maximum horizontal compressive stress vector (S_H_) was further calculated using the complete stress tensor based on Lund and Townend [82].

## Supplementary Material

nwae428_Supplemental_File

## References

[1] Owens TJ, Zandt G. Implications of crustal property variations for models of Tibetan plateau evolution. Nature 1997; 387: 37–43. 10.1038/387037a0

[2] Dewey JF, Burke KC. Tibetan, Variscan, and Precambrian basement reactivation: products of continental collision. J Geol 1973; 81: 683–92. 10.1086/627920

[3] Tapponnier P, Peltzer G, Le Dain YA et al. Propagating extrusion tectonics in Asia: new insights from simple experiments with plasticine. Geology 1982; 10: 611–6. 10.1130/0091-7613(1982)10<611:PETIAN>2.0.CO;2

[4] Bird P . Continental delamination and the Colorado plateau. J Geophys Res Solid Earth 1979; 84: 7561–71. 10.1029/JB084iB13p07561

[5] Davies JH, von Blanckenburg F. Slab breakoff: a model of lithosphere detachment and its test in the magmatism and deformation of collisional orogens. Earth Planet Sci Lett 1995; 129: 85–102. 10.1016/0012-821X(94)00237-S

[6] Royden LH, Burchfiel BC, King RW et al. Surface deformation and lower crustal flow in eastern Tibet. Science 1997; 276: 788–90. 10.1126/science.276.5313.788 9115202

[7] England PC, Houseman G. The mechanics of the Tibetan Plateau. Philos Trans R Soc Lond Ser A-Math Phys Eng Sci 1988; 326: 301–20. 10.1098/rsta.1988.0089

[8] Su T, Farnsworth A, Spicer R et al. No high Tibetan plateau until the Neogene. Sci Adv 2019; 5: eaav2189. 10.1126/sciadv.aav2189 30854430 PMC6402856

[9] Xiong Z, Liu X, Ding L et al. The rise and demise of the Paleogene Central Tibetan Valley. Sci Adv 2022; 8: eabj0944. 10.1126/sciadv.abj0944 35138908 PMC8827648

[10] Fang X, Dupont-Nivet G, Wang C et al. Revised chronology of central Tibet uplift (Lunpola Basin). Sci Adv 2020; 6: eaba7298. 10.1126/sciadv.aba7298 33298435 PMC7725450

[11] Su T, Spicer RA, Wu F-X et al. A Middle Eocene lowland humid subtropical “Shangri-La” ecosystem in central Tibet. Proc Natl Acad Sci USA 2020; 117: 32989–95. 10.1073/pnas.2012647117 33288692 PMC7777077

[12] Kapp P, DeCelles PG. Mesozoic–Cenozoic geological evolution of the Himalayan-Tibetan orogen and working tectonic hypotheses. Am J Sci 2019; 319: 159–254. 10.2475/03.2019.01

[13] Ding L, Xu Q, Yue Y et al. The Andean-type Gangdese Mountains: paleoelevation record from the Paleocene–Eocene Linzhou Basin. Earth Planet Sci Lett 2014; 392: 250–64. 10.1016/j.epsl.2014.01.045

[14] Kapp P, Yin A, Harrison TM et al. Cretaceous-tertiary shortening, basin development, and volcanism in central Tibet. Geol Soc Am Bull 2005; 117: 865–78. 10.1130/B25595.1

[15] Wang C, Zhao X, Liu Z et al. Constraints on the early uplift history of the Tibetan Plateau. Proc Natl Acad Sci USA 2008; 105: 4987–92. 10.1073/pnas.0703595105 18362353 PMC2278176

[16] Deng T, Wang S, Xie G et al. A mammalian fossil from the Dingqing Formation in the Lunpola Basin, northern Tibet, and its relevance to age and paleo-altimetry. Sci Bull 2012; 57: 261–9. 10.1007/s11434-011-4773-8

[17] Sun J, Xu Q, Liu W et al. Palynological evidence for the latest Oligocene–early Miocene paleoelevation estimate in the Lunpola Basin, central Tibet. Palaeogeogr Palaeoclimatol Palaeoecol 2014; 399: 21–30. 10.1016/j.palaeo.2014.02.004

[18] Rowley DB, Currie BS. Palaeo-altimetry of the late Eocene to Miocene Lunpola basin, central Tibet. Nature 2006; 439: 677–81. 10.1038/nature04506 16467830

[19] Tapponnier P, Xu Z, Roger F et al. Oblique stepwise rise and growth of the Tibet Plateau. Science 2001; 294: 1671–7. 10.1126/science.105978 11721044

[20] Taylor M, Peltzer G. Current slip rates on conjugate strike-slip faults in central Tibet using synthetic aperture radar interferometry. J Geophys Res Solid Earth 2006; 111: B12402. 10.1029/2005JB004014

[21] Ratschbacher L, Krumrei I, Blumenwitz M et al. Rifting and strike-slip shear in central Tibet and the geometry, age and kinematics of upper crustal extension in Tibet. Geol Soc London Spec Publ 2011; 353: 127–63. 10.1144/SP353

[22] Armijo R, Tapponnier P, Han T. Late Cenozoic right-lateral strike-slip faulting in southern Tibet. J Geophys Res Solid Earth 1989; 94: 2787–838. 10.1029/JB094iB03p02787

[23] Taylor M, Yin A, Ryerson FJ et al. Conjugate strike-slip faulting along the Bangong-Nujiang suture zone accommodates coeval east-west extension and north-south shortening in the interior of the Tibetan Plateau. Tectonics 2003; 22: 1044. 10.1029/2002TC001361

[24] Yin A, Harrison TM. Geologic evolution of the Himalayan-Tibetan orogen. Annu Rev Earth Planet Sci 2000; 28: 211–80. 10.1146/annurev.earth.28.1.211

[25] Nie S, Tian X, Liang X et al. Pn uppermost mantle tomography of Central Tibet: implication for mechanisms of NS rifts and conjugate faults. Tectonophysics 2020; 788: 228499. 10.1016/j.tecto.2020.228499

[26] Huang S, Yao H, Lu Z et al. High-resolution 3-D shear wave velocity model of the Tibetan plateau: implications for crustal deformation and porphyry Cu deposit formation. J Geophys Res Solid Earth 2020; 125: e2019JB019215. 10.1029/2019JB019215

[27] Wu C, Tian X, Xu T et al. Upper-crustal anisotropy of the conjugate strike-slip fault zone in Central Tibet analyzed using local earthquakes and shear-wave splitting. Bull Seism Soc Am 2019; 109: 1968–84. 10.1785/0120180333

[28] Zhou B, Liang X, Lin G et al. Upper crustal weak zone in central Tibet: an implication from three-dimensional seismic velocity and attenuation tomography results. J Geophys Res Solid Earth 2019; 124: 4654–72. 10.1029/2018JB016653

[29] Dong H, Wei W, Jin S et al. Shaping the surface deformation of central and south Tibetan Plateau: insights from magnetotelluric array data. J Geophys Res Solid Earth 2020; 125: e2019JB019206. 10.1029/2019JB019206

[30] Huang WC, Ni JF, Tilmann F et al. Seismic polarization anisotropy beneath the central Tibetan Plateau. J Geophys Res Solid Earth 2000; 105: 27979–89. 10.1029/2000JB900339

[31] Solon KD, Jones AG, Nelson KD et al. Structure of the crust in the vicinity of the Banggong-Nujiang suture in central Tibet from INDEPTH magnetotelluric data. J Geophys Res Solid Earth 2005; 110: B10102. 10.1029/2003JB002405

[32] Chung S-L, Chu M-F, Zhang Y et al. Tibetan tectonic evolution inferred from spatial and temporal variations in post-collisional magmatism. Earth-Sci Rev 2005; 68: 173–96. 10.1016/j.earscirev.2004.05.001

[33] Wang Q, Wyman DA, Xu J et al. Eocene melting of subducting continental crust and early uplifting of central Tibet: evidence from central-western Qiangtang high-K calc-alkaline andesites, dacites and rhyolites. Earth Planet Sci Lett 2008; 272: 158–71. 10.1016/j.epsl.2008.04.034

[34] Ding L, Kapp P, Yue Y et al. Postcollisional calc-alkaline lavas and xenoliths from the southern Qiangtang terrane, central Tibet. Earth Planet Sci Lett 2007; 254: 28–38. 10.1016/j.epsl.2006.11.019

[35] Zeng Y-C, Xu J-F, Li M-J et al. Late Eocene two-pyroxene trachydacites from the southern Qiangtang Terrane, central Tibetan Plateau: high-temperature melting of overthickened and dehydrated lower crust. J Petrol 2021; 62: egab080. 10.1093/petrology/egab080

[36] Gamond JF . Bridge structures as sense of displacement criteria in brittle fault zones. J Struct Geol 1987; 9: 609–20. 10.1016/0191-8141(87)90146-5

[37] Willemse EJ, Peacock DC, Aydin A. Nucleation and growth of strike-slip faults in limestones from Somerset, UK. J Struct Geol 1997; 19: 1461–77. 10.1016/S0191-8141(97)00056-4

[38] Yin A, Taylor M. Mechanics of V-shaped conjugate strike-slip faults and the corresponding continuum mode of continental deformation. Geol Soc Am Bull 2011; 123: 1798–821. 10.1130/B30159.1

[39] Tunini L, Jiménez-Munt I, Fernandez M et al. Geophysical-petrological model of the crust and upper mantle in the India-Eurasia collision zone. Tectonics 2016; 35: 1642–69. 10.1002/2016TC004161

[40] Hirn A, Jiang M, Sapin M et al. Seismic anisotropy as an indicator of mantle flow beneath the Himalayas and Tibet. Nature 1995; 375: 571–4. 10.1038/375571a0

[41] Gao R, Chen C, Lu Z et al. New constraints on crustal structure and Moho topography in Central Tibet revealed by SinoProbe deep seismic reflection profiling. Tectonophysics 2013; 606: 160–70. 10.1016/j.tecto.2013.08.006

[42] Hirn A, Nercessian A, Sapin M et al. Lhasa block and bordering sutures—a continuation of a 500-km Moho traverse through Tibet. Nature 1984; 307: 25–7. 10.1038/307025a0

[43] Tian X, Wu Q, Zhang Z et al. Joint imaging by teleseismic converted and multiple waves and its application in the INDEPTH-III passive seismic array. Geophys Res Lett 2005; 32: L21315. 10.1029/2005GL023686

[44] DeCelles PG, Kapp P, Ding L et al. Late cretaceous to middle tertiary basin evolution in the central Tibetan Plateau: changing environments in response to tectonic partitioning, aridification, and regional elevation gain. Geol Soc Am Bull 2007; 119: 654–80. 10.1130/B26074.1

[45] Jia G, Bai Y, Ma Y et al. Paleoelevation of Tibetan Lunpola basin in the Oligocene–Miocene transition estimated from leaf wax lipid dual isotopes. Glob Planet Change 2015; 126: 14–22. 10.1016/j.gloplacha.2014.12.007

[46] Wu F, Miao D, Chang M-m *et al*. Fossil climbing perch and associated plant megafossils indicate a warm and wet central Tibet during the late Oligocene. Sci Rep 2017; 7: 878. 10.1038/s41598-017-00928-9 28408764 PMC5429824

[47] Li L, Lu H, Garzione C et al. Cenozoic paleoelevation history of the Lunpola Basin in Central Tibet: new evidence from volcanic glass hydrogen isotopes and a critical review. Earth-Sci Rev 2022; 231: 104068. 10.1016/j.earscirev.2022.104068

[48] Hu X, Ma A, Xue W et al. Exploring a lost ocean in the Tibetan Plateau: birth, growth, and demise of the Bangong-Nujiang Ocean. Earth-Sci Rev 2022; 229: 104031. 10.1016/j.earscirev.2022.104031

[49] The Geological Survey of Jilin Province. Geological map of the Duoba sheet (H45C001004). Scale 1: 250000. Beijing: Geological Publishing House, 2003.

[50] The Geological Survey of Jilin Province. Geological map of the Angdaercuo sheet (I45C004004). Scale 1: 250000. Beijing: Geological Publishing House, 2006.

[51] Chew D, O'Sullivan G, Caracciolo L et al. Sourcing the sand: accessory mineral fertility, analytical and other biases in detrital U-Pb provenance analysis. Earth-Sci Rev 2020; 202: 103093. 10.1016/j.earscirev.2020.103093

[52] Flowers RM, Ketcham RA, Shuster DL et al. Apatite (U–Th)/He thermochronometry using a radiation damage accumulation and annealing model. Geoch Cos acta 2009; 73: 2347–65. 10.1016/j.gca.2009.01.015

[53] Reiners PW, Farley KA. Influence of crystal size on apatite (U–Th)/He thermochronology: an example from the Bighorn Mountains, Wyoming. Earth Planet Sci Lett 2001; 188: 413–20. 10.1016/S0012-821X(01)00341-7

[54] Rohrmann A, Kapp P, Carrapa B et al. Thermochronologic evidence for plateau formation in central Tibet by 45 ma. Geology 2012; 40: 187–90. 10.1130/G32530.1

[55] Li C, Zhao Z, Lu H et al. Late Mesozoic-Cenozoic multistage exhumation of the central Bangong-Nujiang Suture, Central Tibet. Tectonophysics 2022; 827: 229268. 10.1016/j.tecto.2022.229268

[56] Jolly RJ, Lonergan L. Mechanisms and controls on the formation of sand intrusions. J Geol Soc 2002; 159: 605–17. 10.1144/0016-764902-025

[57] Sach VJ, Buchner E, Schmieder M. Enigmatic earthquake-generated large-scale clastic dyke in the Biberach area (SW Germany). Sediment Geol 2020; 398: 105571. 10.1016/j.sedgeo.2019.105571

[58] Flowers R, Zeitler P, Danišík M et al. (U-Th)/He chronology: part 1. Data, uncertainty, and reporting. Geol Soc Am Bull 2022; 135: 104–36. 10.1130/B36266.1

[59] Ding L, Kapp P, Zhong D et al. Cenozoic volcanism in Tibet: evidence for a transition from oceanic to continental subduction. J Petrol 2003; 44: 1833–65. 10.1093/petrology/egg061

[60] Coward MP, Dewey JF, Hancock PL. Continental Extensional Tectonics. Oxford: Blackwell Scientific Publications, 1987.

[61] Mazzoli S, Invernizzi C, Marchegiani L et al. Brittle-ductile shear zone evolution and fault initiation in limestones, Monte Cugnone (Lucania), southern Apennines, Italy. Geol Soc London Spec Publ 2004; 224: 353–73. 10.1144/GSL.SP.2004.224.01.2

[62] Hill DP . A model for earthquake swarms. J Geophys Res Solid Earth 1977; 82: 1347–52. 10.1029/JB082i008p01347

[63] Shaw HR . Fracture mechanisms of magma transport from the mantle to the surface. In: Hardgraves R.B. (ed.). Physics of Magmatic Processes. Princeton: Princeton University Press, 1980, 201–64.

[64] Wang H, Wright TJ, Liu-Zeng J et al. Strain rate distribution in south-central Tibet from two decades of InSAR and GPS. Geophys Res Lett 2019; 46: 5170–9. 10.1029/2019GL081916

[65] Clemens J, Mawer C. Granitic magma transport by fracture propagation. Tectonophysics 1992; 204: 339–60. 10.1016/0040-1951(92)90316-X

[66] Petford N . Dykes or diapirs? Earth Environ Sci Trans R Soc Edinb 1996; 87: 105–14. 10.1017/S0263593300006520

[67] Flesch LM, Holt WE, Silver PG et al. Constraining the extent of crust–mantle coupling in central Asia using GPS, geologic, and shear wave splitting data. Earth Planet Sci Lett 2005; 238: 248–68. 10.1016/j.epsl.2005.06.023

[68] Frost C, Bell J, Frost B et al. Crustal growth by magmatic underplating: isotopic evidence from the northern Sherman batholith. Geology 2001; 29: 515–8. 10.1130/0091-7613(2001)029<0515:CGBMUI>2.0.CO;2

[69] Leloup PH, Arnaud N, Lacassin R et al. New constraints on the structure, thermochronology, and timing of the Ailao Shan-Red River shear zone, SE Asia. J Geophys Res Solid Earth 2001; 106: 6683–732. 10.1029/2000JB900322

[70] Leloup PH, Kienast J-R. High-temperature metamorphism in a major strike-slip shear zone: the Ailao Shan—Red River, People's Republic of China. Earth Planet Sci Lett 1993; 118: 213–34. 10.1016/0012-821X(93)90169-A

[71] Leloup PH, Lacassin R, Tapponnier P et al. The Ailao Shan-Red river shear zone (Yunnan, China), tertiary transform boundary of Indochina. Tectonophysics 1995; 251: 3–84. 10.1016/0040-1951(95)00070-4

[72] Peltzer G, Tapponnier P. Formation and evolution of strike-slip faults, rifts, and basins during the India-Asia collision: an experimental approach. J Geophys Res Solid Earth 1988; 93: 15085–117. 10.1029/JB093iB12p15085

[73] Tapponnier P, Lacassin R, Leloup PH et al. The Ailao Shan/Red River metamorphic belt: tertiary left-lateral shear between Indochina and South China. Nature 1990; 343: 431–7. 10.1038/343431a0

[74] Tapponnier P, Peltzer G, Armijo R. On the mechanics of the collision between India and Asia. Geol Soc London Spec Publ 1986; 19: 113–57. 10.1144/GSL.SP.1986.019.01.07

[75] Zhang B, Cai F, Chen S et al. Sinistral strike-slip shearing along the Jiali shear zone around the Eastern Himalaya syntaxis region: evidences for Oligocene eastward limited translation of Tibet. J Struct Geol 2020; 139: 104136. 10.1016/j.jsg.2020.104136

[76] Replumaz A, Capitanio FA, Guillot S et al. The coupling of Indian subduction and Asian continental tectonics. Gondwana Res 2014; 26: 608–26. 10.1016/j.gr.2014.04.003

[77] Replumaz A, Tapponnier P. Reconstruction of the deformed collision zone between India and Asia by backward motion of lithospheric blocks. J Geophys Res Solid Earth 2003; 108: 2285. 10.1029/2001JB000661

[78] Li S, Advokaat EL, van Hinsbergen DJ et al. Paleomagnetic constraints on the Mesozoic-Cenozoic paleolatitudinal and rotational history of Indochina and South China: review and updated kinematic reconstruction. Earth-Sci Rev 2017; 171: 58–77. 10.1016/j.earscirev.2017.05.007

[79] Yamaji A, Sato K. Clustering of fracture orientations using a mixed Bingham distribution and its application to paleostress analysis from dike or vein orientations. J Struct Geol 2011; 33: 1148–57. 10.1016/j.jsg.2011.05.006

[80] Yamaji A . Genetic algorithm for fitting a mixed Bingham distribution to 3D orientations: a tool for the statistical and paleostress analyses of fracture orientations. Island Arc 2016; 25: 72–83. 10.1111/iar.12135

[81] Baer G, Beyth M, Reches Z. Dikes emplaced into fractured basement, Timna igneous complex, Israel. J Geophys Res Solid Earth 1994; 99: 24039–50. 10.1029/94JB02161

[82] Lund B, Townend J. Calculating horizontal stress orientations with full or partial knowledge of the tectonic stress tensor. Geophys J Int 2007; 170: 1328–35. 10.1111/j.1365-246X.2007.03468.x

