# Supplementary material for: Lithospheric strike-slip faulting in central Tibet since 35–32 Ma and implications for the incipient Asian extrusional tectonics
Source: Natl Sci Rev. 2024 Nov 28;12(2):nwae428. doi: 10.1093/nsr/nwae428 (PMC11761741; doi:10.1093/nsr/nwae428)
Supplement: nwae428_Supplemental_File [file nwae428_supplemental_file.doc]

**Supplementary Data for**

**Lithospheric strike-slip faulting in central Tibet since 35-32 Ma**

Haijian Lu^1, 2^, Haibing Li^1, 2^*, Zhongjin Xiang^1^, Marco G. Malusà^3^, Chunrui Li^1, 2^, Zhiyong Zhang^4^, Lin Wu^4^, Xuxuan Ma^1, 2^, Jiawei Pan^1, 2^

^1^SinoProbe Laboratory, Key Laboratory of Continental Dynamics of Ministry of Natural Resources, Institute of Geology, Chinese Academy of Geological Sciences, Beijing 100037, China;

^2^Jiangsu Donghai Crustal Activity in Deep Holes of the Continental Scientific Drilling National Observation and Research Station, Lianyungang 222300, China;

^3^University of Milano-Bicocca, Milano 20126, Italy;

^4^Institute of Geology and Geophysics, Chinese Academy of Sciences, Beijing 100029, China.

Correspondence to: H. Li, lihaibing06@163.com

**Contents of this file**

Supplementary Data and Methods

Supplmentary Figures 1 to 15

Supplementary References

**Supplementary Data and Methods**

**Zircon U-Pb dating**

The measurement was conducted using laser ablation–inductively coupled plasma–mass spectrometry (Agilent 8900 ICP-QQQ coupled with an ESI New Wave NWR 193UC laser ablation system) at the Beijing Quick-Thermo Science and Technology Co., Ltd. Individual zircon grain mounted and polished in epoxy were ablated in a constant stream of He that is mixed downstream with N2 and Ar before entering the torch region of the ICP–QQQ. Following warmup of the ICP–QQQ and connection with the laser ablation system, the ICPMS is tuned for robust plasma conditions by optimizing laser and ICP–QQQ setting, monitoring ^232^Th^16^O^+^/^232^Th^+^ ratios (always ≤0.2%) and ^238^U^+^/^232^Th^+^ ratios (always between 0.95 and 1.05) while ablating NIST SRM 612 in line scan mode. All age data were obtained by single spot analyses with a spot diameter of 20 μm and a crater depth of approximately 15–20 μm. Background subtraction and correction for laser downhole elemental fractionation were performed using the Iolite data reduction package within the Wavemetrics Igor Pro data analysis software [1]. Concordia diagrams (Wetherill and Tera-Wasserburg) were processed based on ISOPLOT 4.15. We use ^206^Pb/^238^U age when the sample age is younger than 1000 Ma and ^207^Pb/^235^U age when the sample age is older than 1000 Ma. Age uncertainties are reported as the 2σ standard error of replicate analyses.

**Apatite U-Th/He dating**

Each grain was subsequently wrapped in a 1 mm × 1 mm platinum (Pt) capsule and loaded on a drilled oxygen-free copper disk. Helium measurement was conducted using fully automatic helium extraction system called Alphachron MK II (Australian Scientific Instrument Pty Limited). Following helium extraction, Pt wrapped grains were moved to Savillex PFA vials and spiked with ^230^Th-^235^U solution with known concentration. All the spiked solutions were analyzed on a Thermo Fisher X-Series II inductively coupled plasma mass spectrometry (ICP-MS). Age calculation was processed by a java-based program named Helioplot [2] and corrected for alpha emission based on the procedure of Gautheron et al. [3]. MK-1 apatite [4] was applied as a reference standard to verify the analytical procedure. Age uncertainties are reported as the 2σ standard error of replicate analyses.

**Whole-rock geochemistry**

About 40 mg powder for each sample was mixed with 0.5ml 60wt% HNO_3_ and 1.0ml 40% HF in high-pressure polytetrafluoroethylene (PTFE) bombs. These bombs were then steel-jacketed and placed in the oven at 195℃ for 3 days to ensure complete digestion. After cooling, the bombs were dried down on a hotplate and re-dissolved with 5ml 15wt% HNO_3_ and 1ml Rh internal standard, then sealed and placed in the oven at 150℃ overnight. An aliquot of the digestions (dilution factor 2000) was nebulized into Agilent Technologies 7700x quadrupole ICP-MS (Tokyo, Japan) to determine trace elements, while another aliquot (dilution factor 500) was introduced into Agilent Technologies 5110 ICP-OES (Penang, Malaysia) to determine major and minor elements except Si.

Subsequently, about 30 mg powder for each sample was mixed with 150 mg NaOH in silver crucible. These crucibles were put in the muffle furnace at 650℃ for 20 minutes to ensure complete decomposition. Fusion products were neutralized by HCl and an aliquot of the digestions (dilution factor 250) was introduced into Agilent 5110 ICP-OES to measure Si.

**Whole-rock Sr-Nd isotopic measurement**

About 40 mg powder for each sample was mixed with 0.5ml 60wt% HNO3 and 1.0ml 40wt% HF in high-pressure PTFE bombs. These bombs were steel-jacketed and placed in the oven at 195℃ for 3 days. Digested samples were dried down on a hotplate and reconstituted in 1.5ml of 1.5 N HCl before ion exchange purification. Sr and Nd isotopes were purified from the same digestion solution by a two-step column chemistry. In the first step of exchange column, Bio-Rad AG 50W-X8 and Eichrom Sr-Spec resins were used to separate Sr and REEs from the sample matrix. Subsequently, Nd was separated from other REEs with Teflon powder coated in Eichrom Ln-Spec resin. The Sr- and Nd-bearing elutions were dried and redissolved in 1.0 ml 2 wt% HNO_3_. The measurement of small aliquots of each solution was conducted by an Agilent Technologies 7700x quadrupole ICP-MS to determine the contents of Sr and Nd. Then we introduced Diluted solutions (50 ppb Sr, 50 ppb Nd, doped with 10 ppb Tl for both) into a Nu Instruments Nu Plasma II MC-ICP-MS through a Teledyne Cetac Technologies Aridus II desolvating nebulizer system.


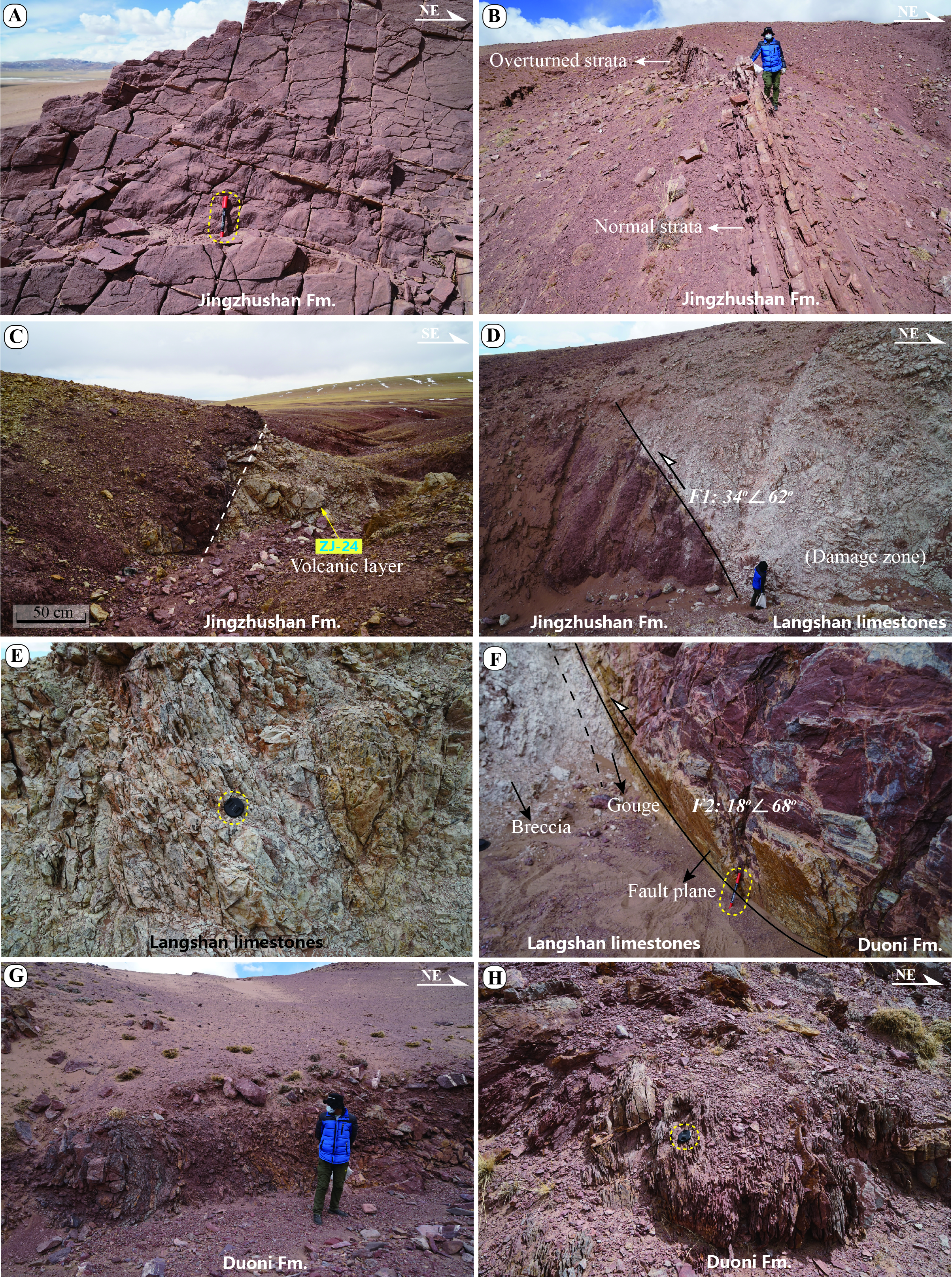


**Fig. S1**. Representative field photos of the Jingzhushan (A-D), Langshan (D-F), and Duoni Fms (F-H). Note the thrust contacts among the Jingzhushan, Langshan, and Duoni Fms (D, F).


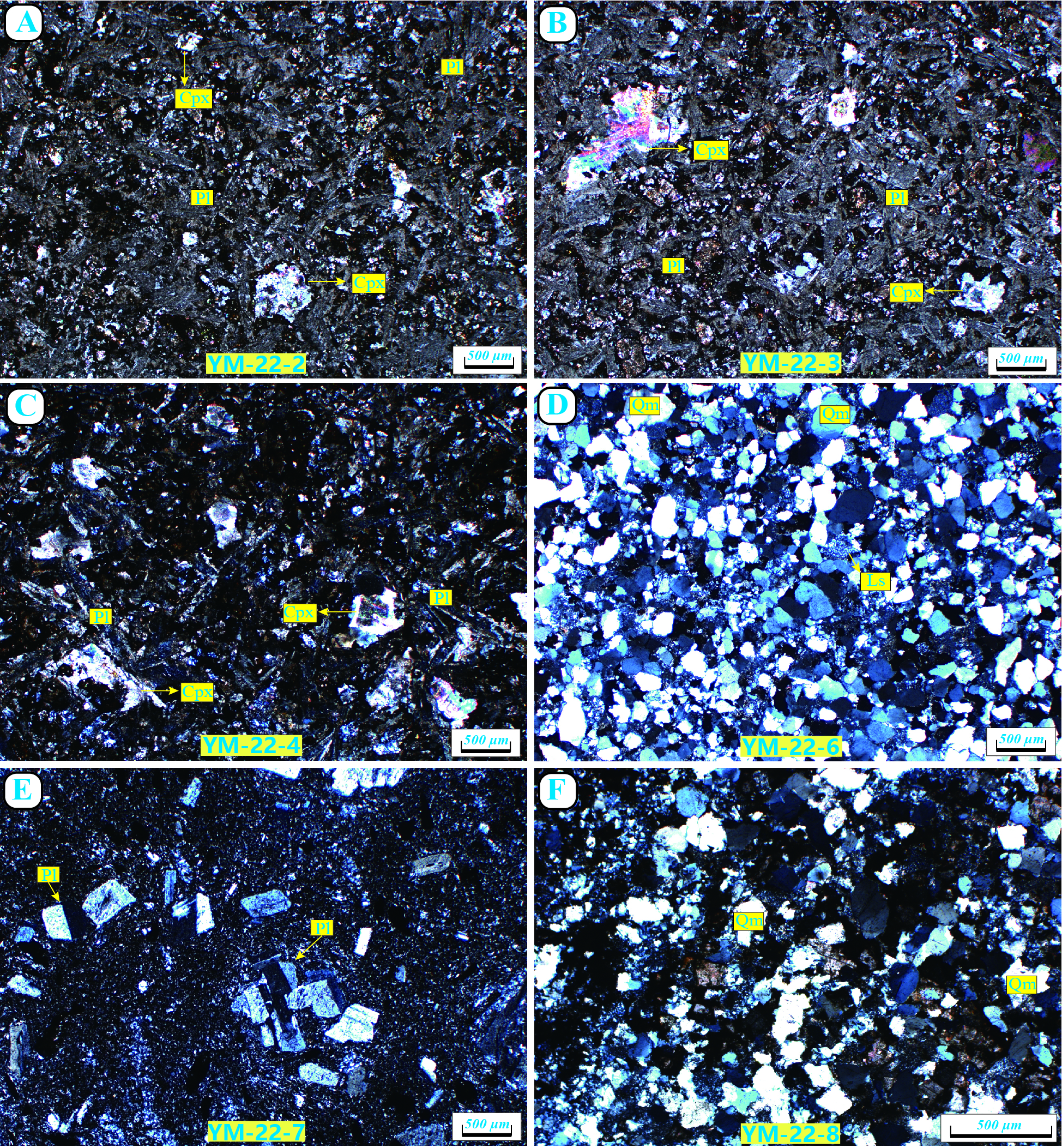


**Fig. S2**. Photomicrographs of the diabase (YM-22-2, YM-22-3 and YM-22-4) and dacite porphyry (YM-22-7) and clastic (YM-22-6 and YM-22-8) dykes. Abbreviations are as follows: Cpx, clinopyroxene; Pl, plagioclase; Qm, monocrystalline quartz; Ls, sedimentary lithic.


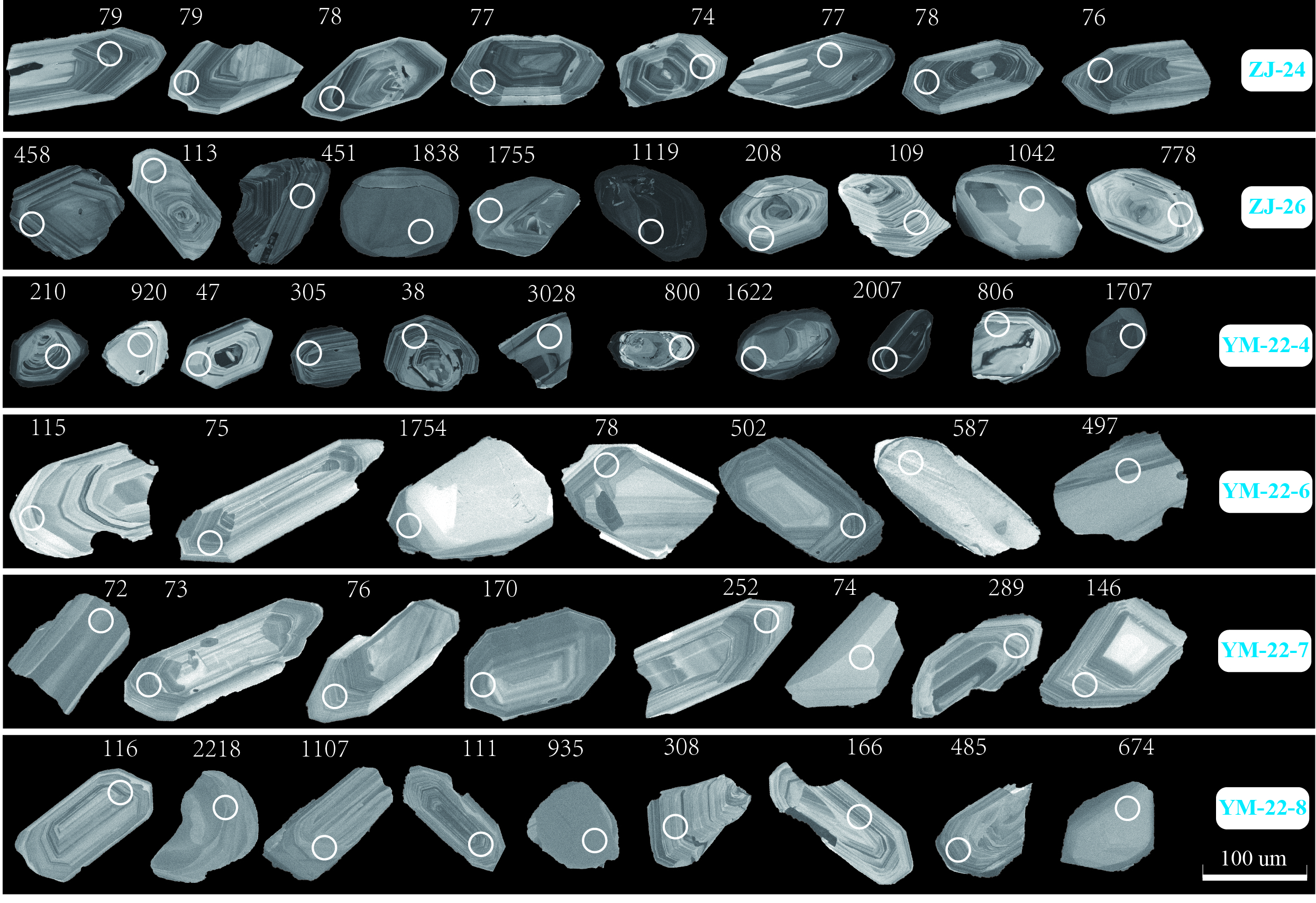


**Fig. S3**. Representative cathodoluminescence (CL) images of zircon grains from the interbedded volcanic (ZJ-24), sandstone (ZJ-26), diabase (YM-22-2, YM-22-3 and YM-22-4), dacite porphyry (YM-22-7) and clastic (YM-22-6 and YM-22-8) dyke samples.


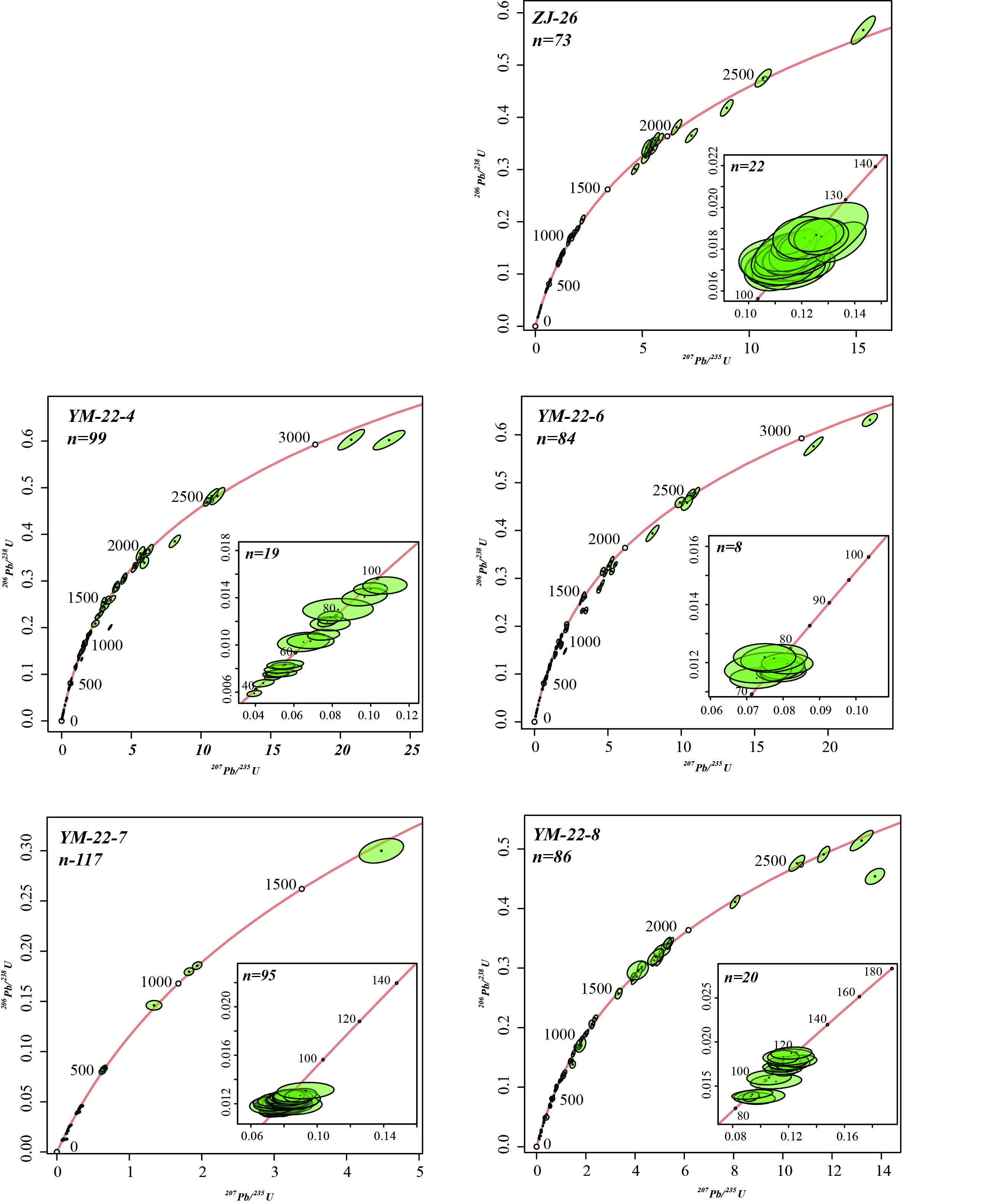


**Fig. S4**. The U-Pb age Concordia diagrams of the sandstone (ZJ-26), diabase (YM-22-4), dacite porphyry (YM-22-7) and clastic (YM-22-6 and YM-22-8) dyke samples. Inset map on the lower right indicates the youngest population.


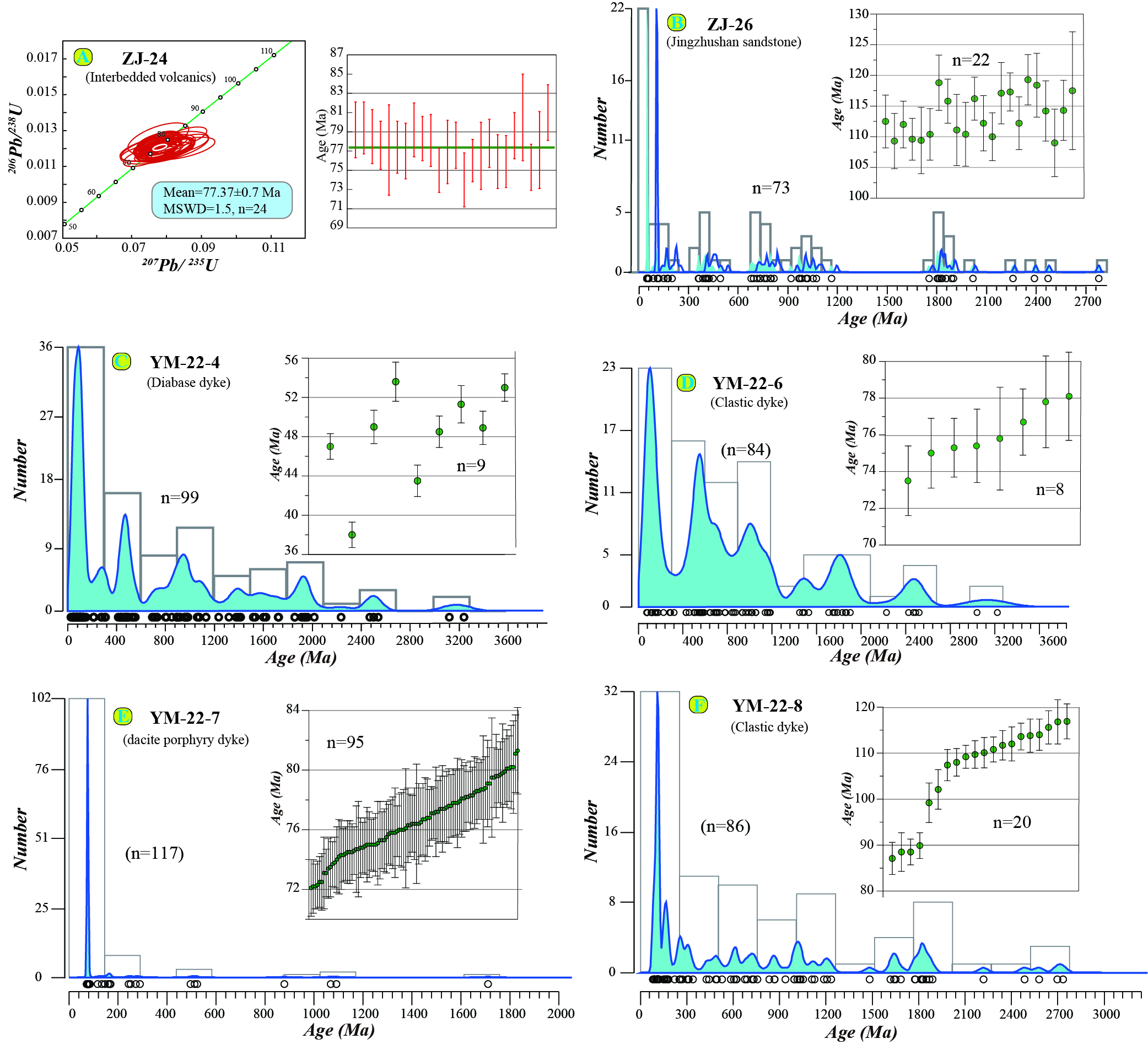


**Fig. S5.** Zircon U-Pb dating results of the interbedded volcanics (ZJ-24), sandstone (ZJ-26), diabase (YM-22-4), dacite porphyry (YM-22-7) and clastic dyke (YM-22-6 and YM-22-8) samples. The inset on the upper right (B-F) of each plot shows the youngest age population.


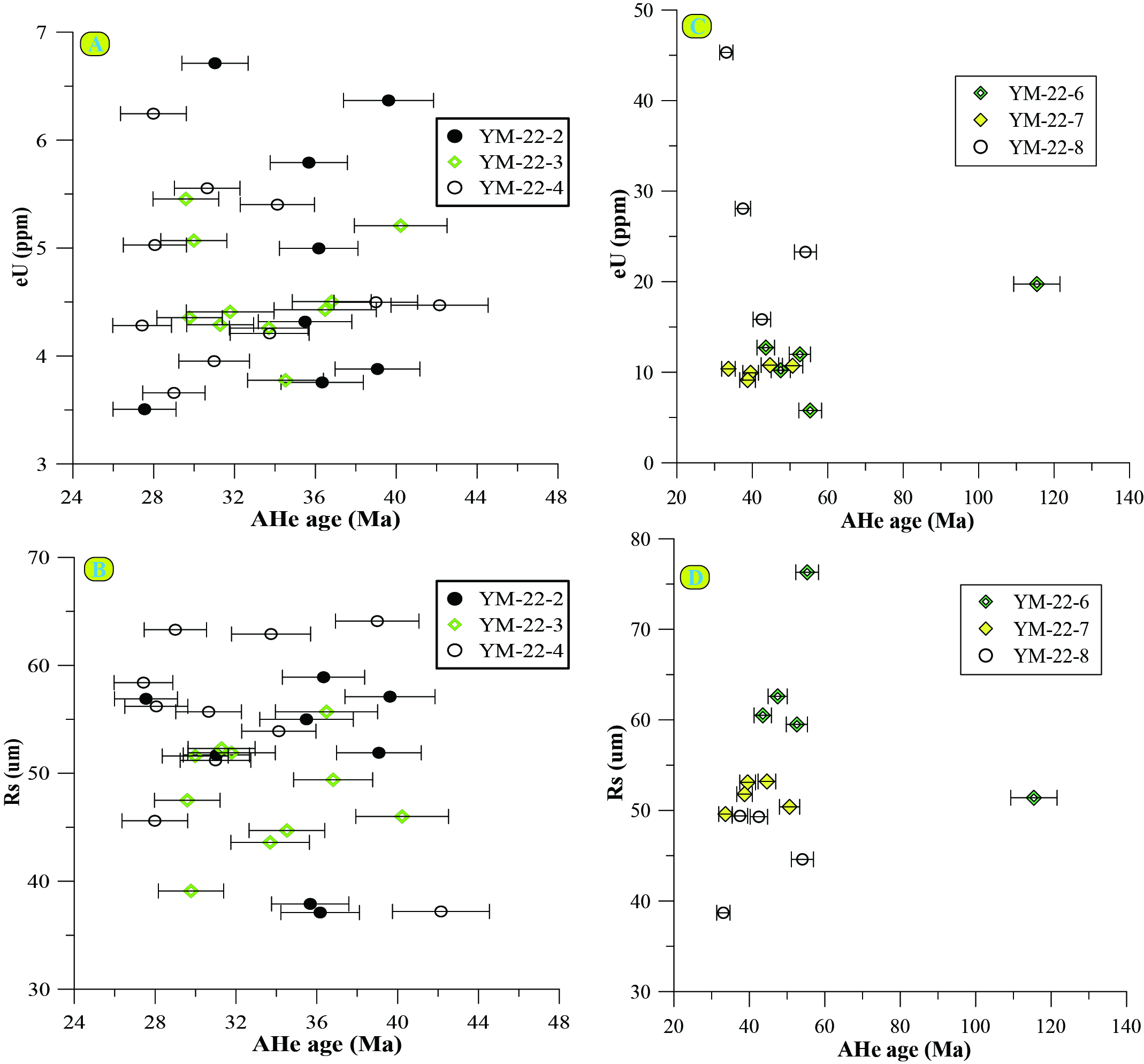


**Fig. S6.** Relationships of corrected AHe age against effective uranium (eU; A, C) and against spherical equivalent radius (Rs; B, D) of the diabase (YM-22-2, YM-22-3 and YM-22-4), dacite porphyry (YM-22-7) and clastic (YM-22-6 and YM-22-8) dyke samples.


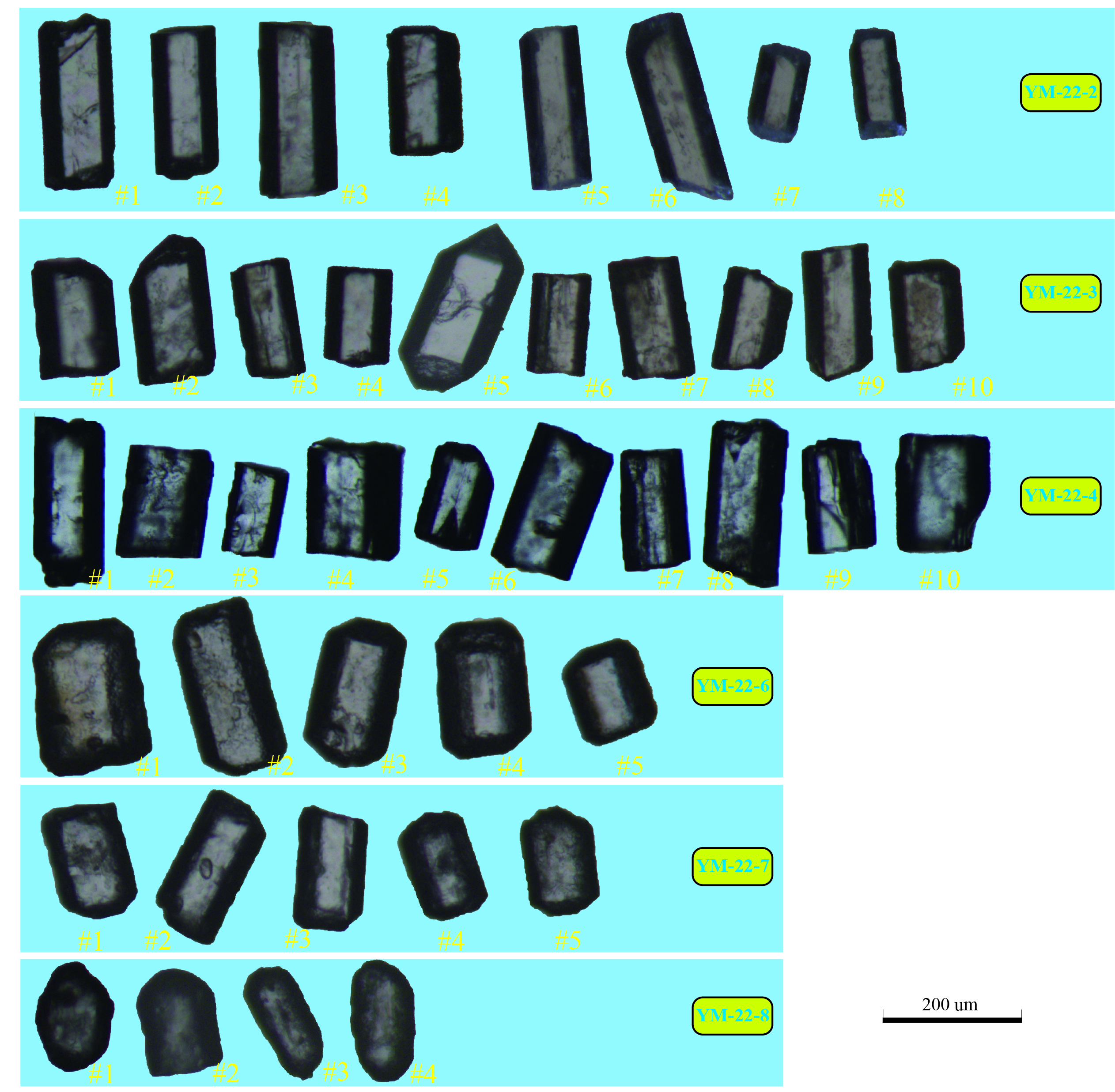


**Fig. S7**. The morphology of the dated apatite grains of the diabase (YM-22-2, YM-22-3 and YM-22-4), dacite porphyry (YM-22-7) and clastic (YM-22-6 and YM-22-8) dyke samples.


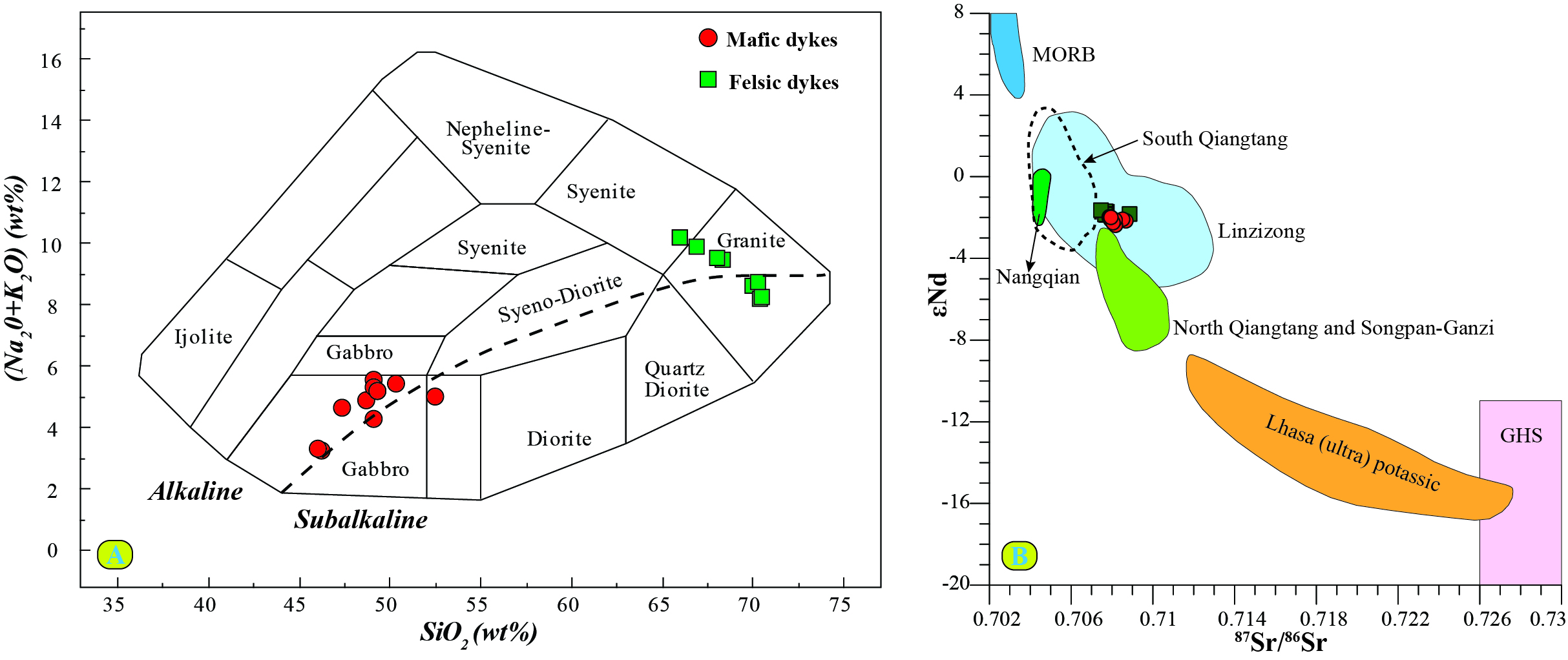


**Fig. S8**. Total-alkalis–silica (A) and εNd(t) versus (87Sr/86Sr)i (B) diagrams for the mafic (YM-22-2, YM-22-3 and YM-22-4) and felsic (YM-22-7) dyke samples in the Lunpola Basin. Fields for various magmatic rocks are adapted from Ding et al. [5].


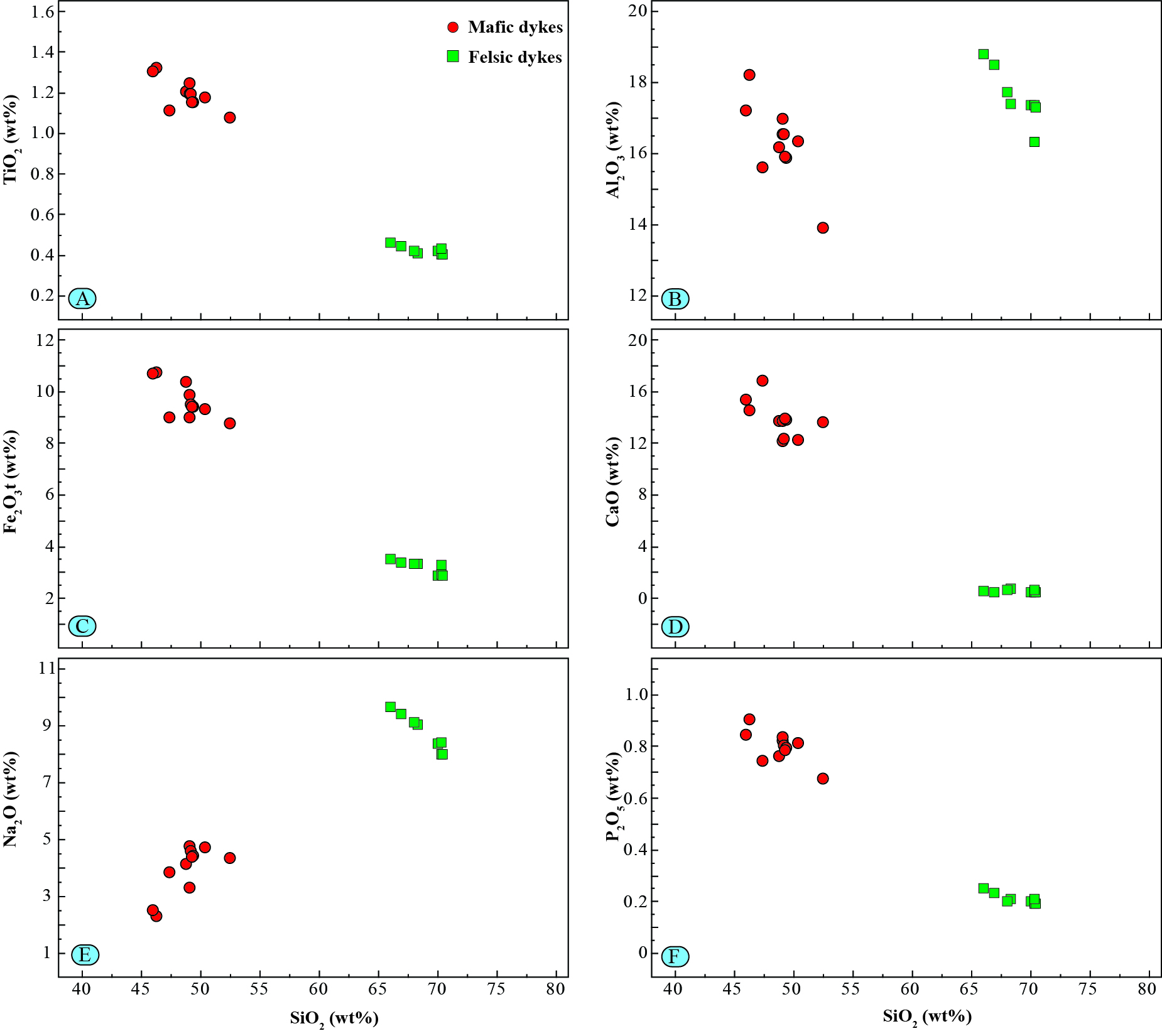


**Fig. S9**. Harker diagrams of selected major and trace elements for the mafic (YM-22-2, YM-22-3 and YM-22-4) and felsic (YM-22-7) dykes in the Lunpola Basin.


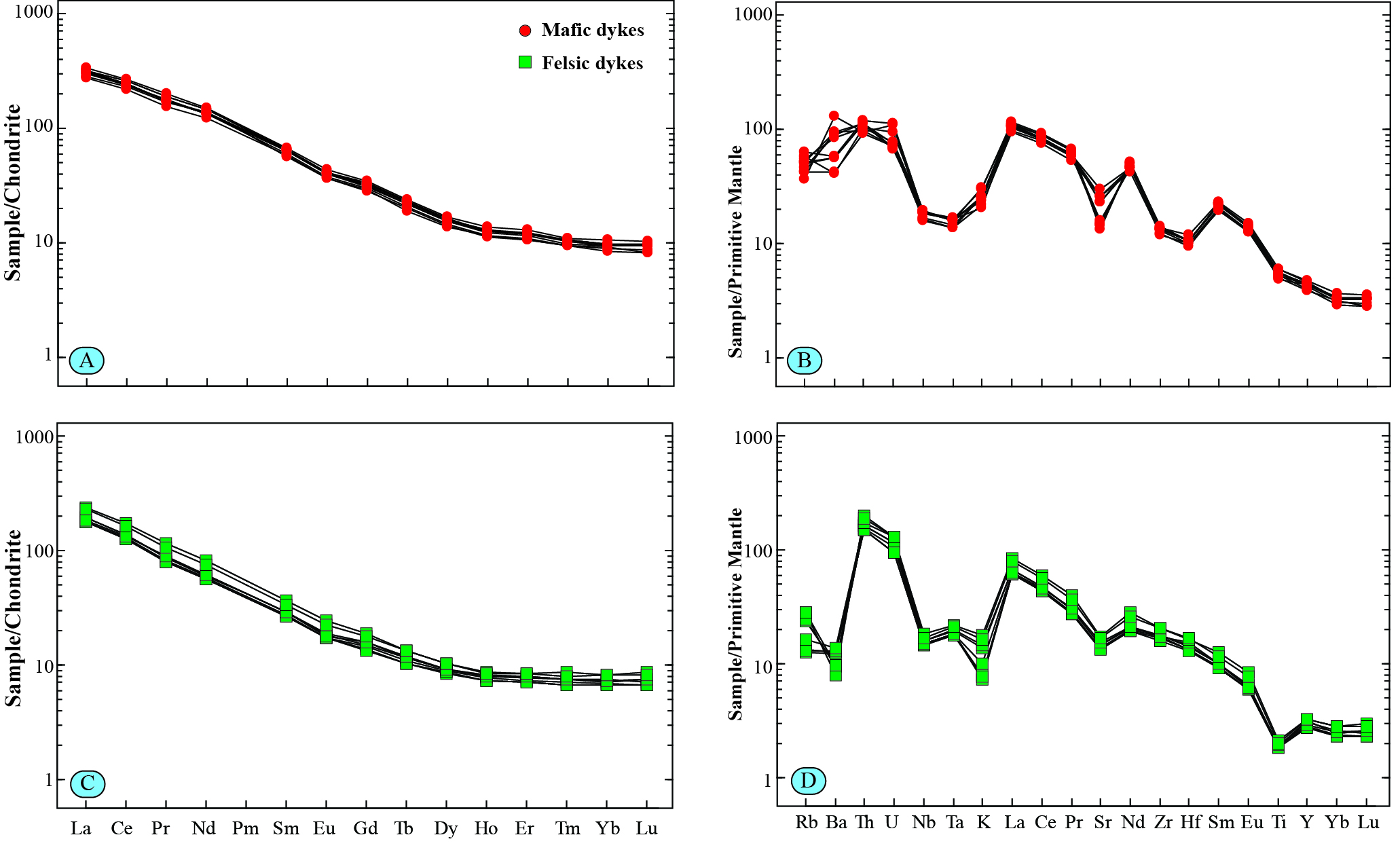


**Fig. S10.** Chondrite-normalized REE plots (A, C) and primitive-mantle normalized

trace-element variation plots (B, D) for the mafic (YM-22-2, YM-22-3 and YM-22-4) and felsic (YM-22-7) dykes in the Lunpola Basin. Normalizing values are from Sun and McDonough [6].


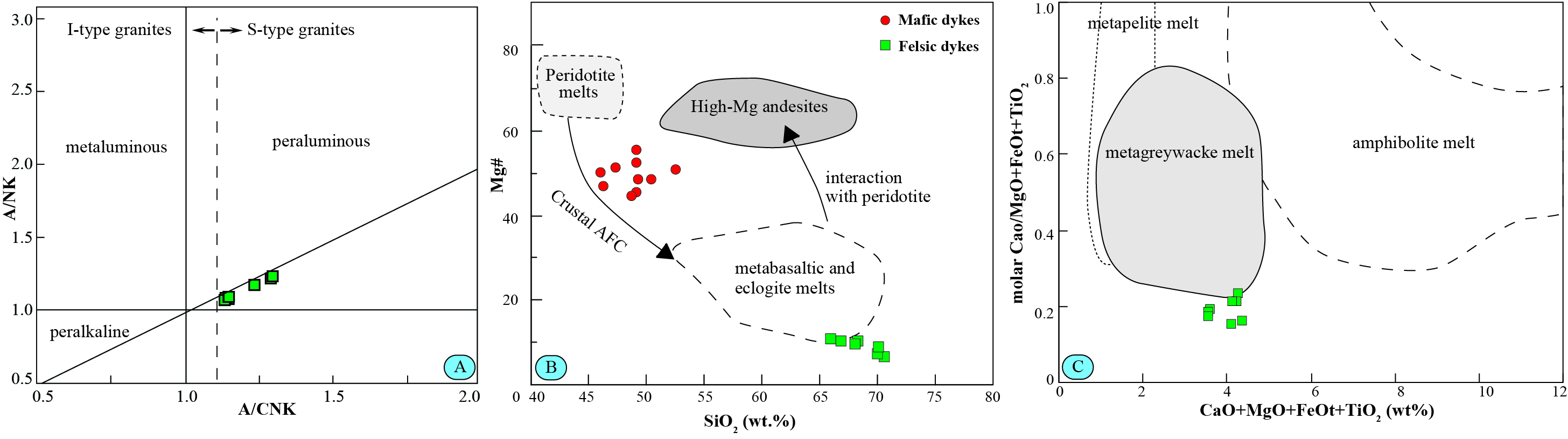


**Fig. S11.** Plot of A/NK vs. A/CNK (A) and Harker diagrams of Mg# values vs. SiO_2_ and molar CaO/MgO+FeO+TiO_2_ vs. CaO+MgO+FeOt+TiO_2_ for the mafic (YM-22-2, YM-22-3 and YM-22-4) and felsic (YM-22-7) dykes in the Lunpola Basin.


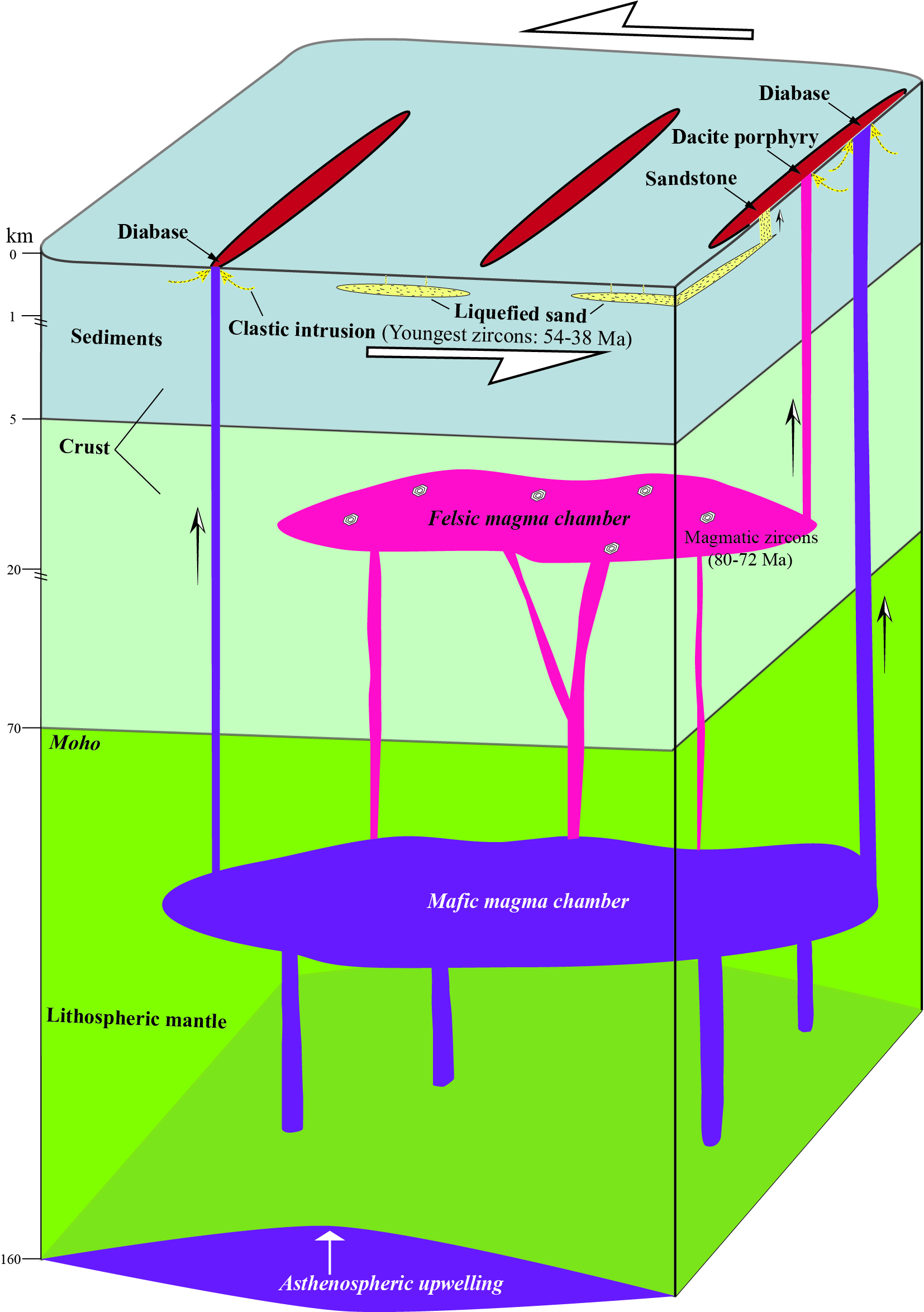
**Fig. S12**. Schematic illustration of coincident intrusion of the diabase, dacite porphyry and clastic dykes following shear fracture opening at 35-32 Ma.


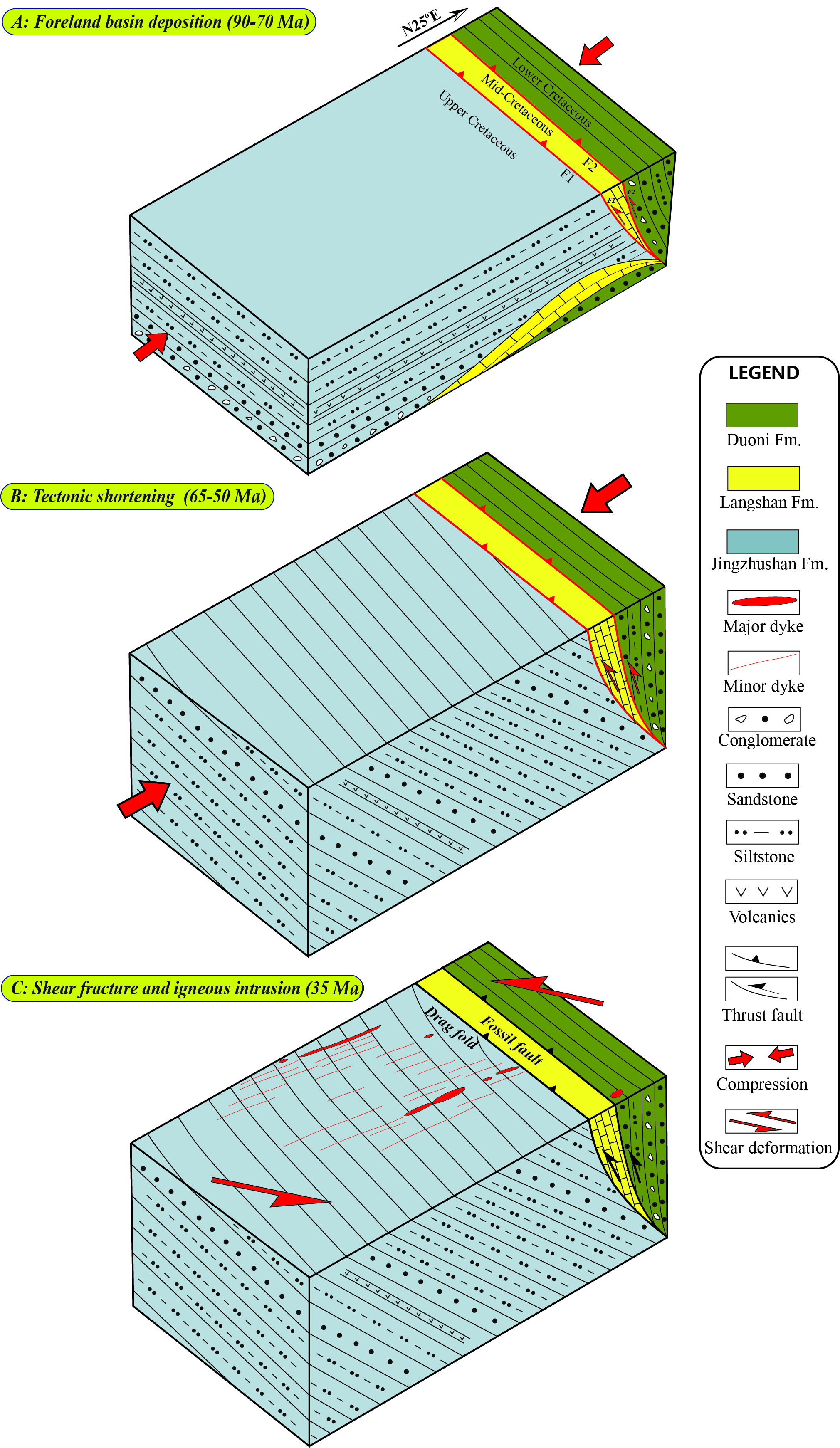


**Fig. S13**. Three-stage tectonic, sedimentary, and magmatic evolution of the study area since late Cretaceous.


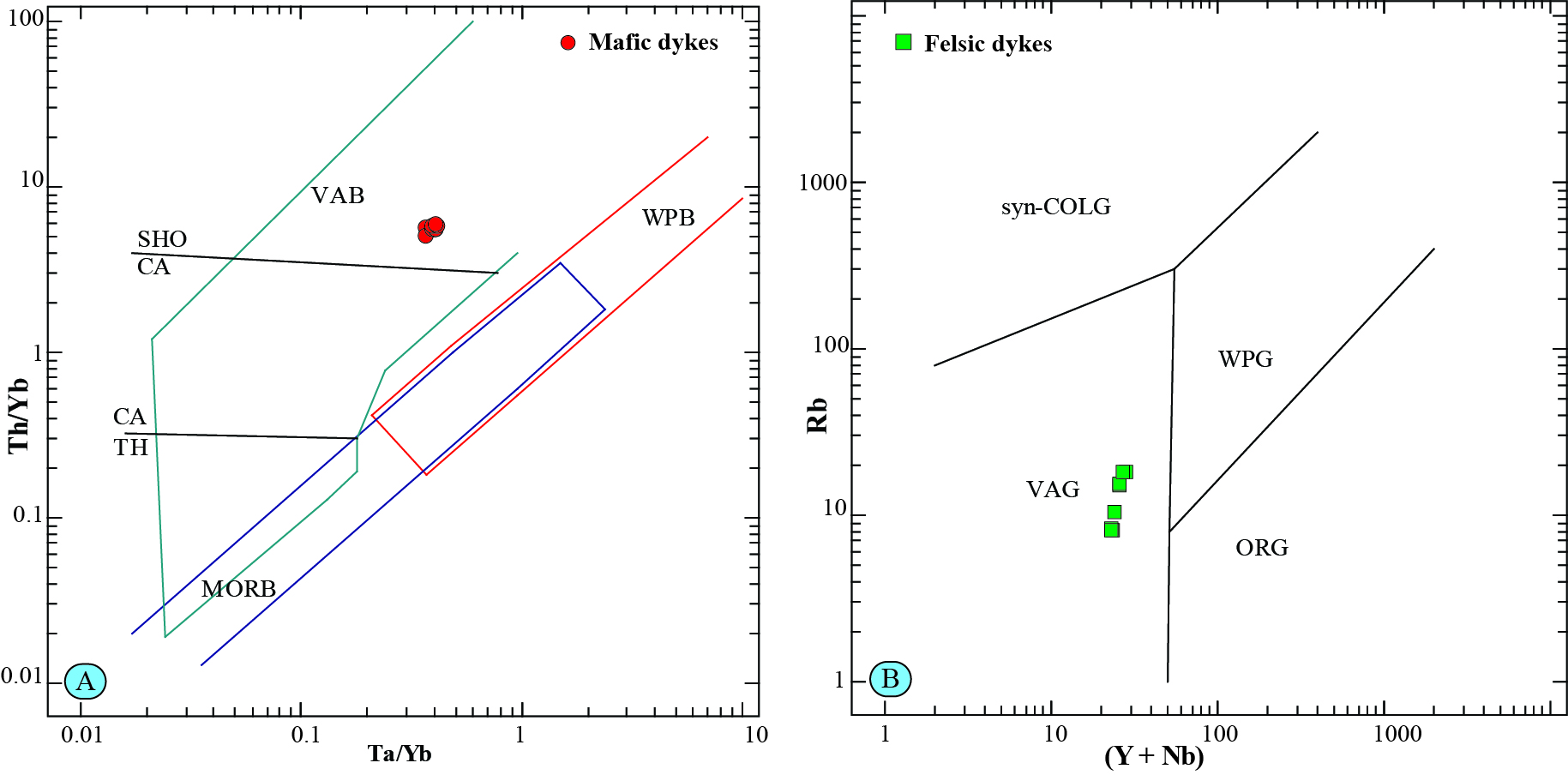


**Fig. S14.** Plots of Th/Yb vs. Ta/Yb (A) and Rb vs. Y+Nb (B) for the mafic (YM-22-2, YM-22-3 and YM-22-4) and felsic (YM-22-7) dykes in the Lunpola Basin.


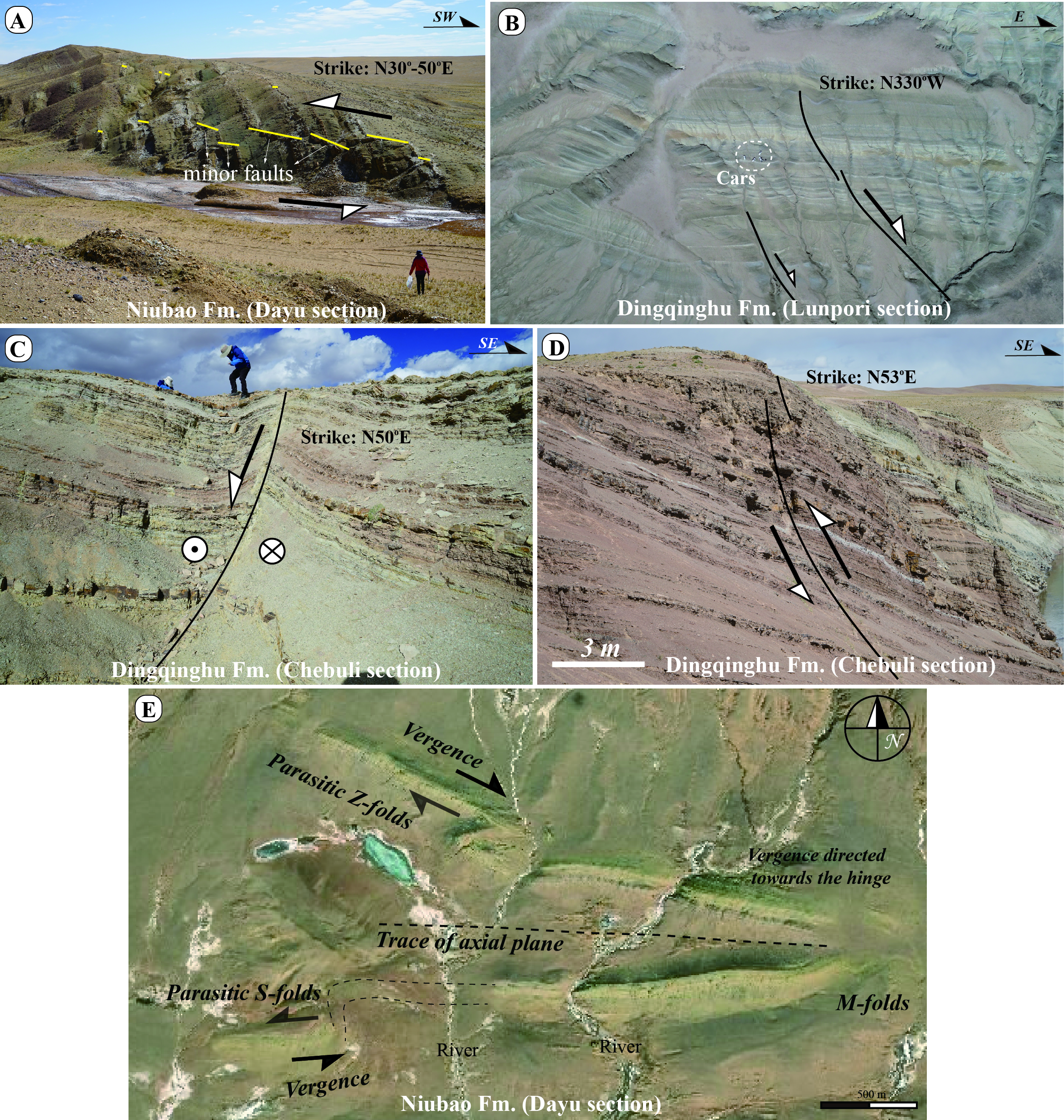


**Fig. S15**. The dominant strike-slip structures (northwest-striking right-lateral and northeast-striking left-lateral) in the Niubao and Dingqinghu Fms of the Lunpola basin, central Tibet. Please refer to Fig. 2 for the locations of the sedimentary sections (Dayu, Lunpori, and Chebuli).

**Supplementary References**

1. Paton C, Woodhead JD, Hellstrom JC et al. Improved laser ablation U‐Pb zircon geochronology through robust downhole fractionation correction. *Geochem, Geophys, Geosyst* 2010; **11**.

2. Vermeesch P. HelioPlot, and the treatment of overdispersed (U–Th–Sm)/He data. *Chem Geology* 2010; **271**: 108-111.

3. Gautheron C, Tassan-Got L, Barbarand J et al. Effect of alpha-damage annealing on apatite (U–Th)/He thermochronology. *Chem Geology* 2009; **266**: 157-170.

4. Wu L, Shi G, Danišík M et al. MK‐1 Apatite: A new potential reference material for (U‐Th)/He dating. *Geostand Geoanal Res* 2019; **43**: 301-315.

5. Ding L, Kapp P, Yue Y et al. Postcollisional calc-alkaline lavas and xenoliths from the southern Qiangtang terrane, central Tibet. *Earth Planet Sci Lett* 2007; **254**: 28-38.

6. Sun S-S, McDonough WF. Chemical and isotopic systematics of oceanic basalts: implications for mantle composition and processes. *Geol Soc, London, Special Publications* 1989; **42**: 313-345.
